# Phase II trial protocol of focal prostate ablation combined with androgen deprivation therapy for prostate cancer treatment

**DOI:** 10.1371/journal.pone.0337828

**Published:** 2025-12-16

**Authors:** Jason Koehler, Daniel Lama, Megan Mendez, Wei-Wen Hsu, Aytekin Oto, Russell Szmulewitz, Abhinav Sidana

**Affiliations:** 1 University of Cincinnati College of Medicine, Cincinnati, Ohio, United States of America; 2 Department of Urology, University of Cincinnati, Cincinnati, Ohio, United States of America; 3 Section of Urology, Department of Surgery, University of Chicago, Chicago, Illinois, United States of America; 4 Division of Biostatistics and Bioinformatics, University of Cincinnati College of Medicine, Cincinnati, Ohio, United States of America; 5 Department of Radiology, University of Chicago, Chicago, Illinois, United States of America; 6 Section of Hematology/Oncology, Department of Internal Medicine, University of Chicago, Chicago, Illinois, United States of America; University of Health and Allied Sciences, GHANA

## Abstract

**Background:**

Prostate cancer (PCa) is one of the most commonly diagnosed cancers. Treatments for PCa with less adverse effects than whole gland interventions, such as focal therapy (FT), often involve a higher risk of PCa recurrence. Combining FT with other treatments could increase the efficacy while maintaining a low side effect profile. This study aims to determine the proportion of residual/recurrent clinically significant PCa following the combination treatment of three months of androgen deprivation therapy (ADT) and FT of the prostate, as well as the safety of this treatment regimen.

**Methods:**

This study will be a single arm phase II trial with a recruitment goal of 57 patients with treatment naïve non-metastatic intermediate risk PCa. Patients will complete the I-PSS, SHIM, and EPIC-26 questionnaires at the screening visit. Patients will then comply with a three-month treatment course of ADT. Eight weeks after beginning ADT, patients will undergo FT of the prostate via high intensity focused ultrasound or cryoablation. Follow up visits will occur every three months for a year post FT to monitor for side effects, repeat questionnaires, perform a clinical assessment, and obtain PSA and testosterone values. Twelve months after FT, a surveillance multiparametric MRI and MRI-targeted biopsy will be performed to assess for treatment failure.

**Discussion:**

The primary endpoint of this trial is to determine the proportion of men with clinically significant PCa as evaluated by a surveillance mpMRI and MRI-TB at 12-months following FT. If successful, this treatment approach could offer a new option for treatment with fewer side effects than whole gland interventions and more efficacy than FT alone. Furthermore it could inform the need for further research into multimodal treatment options for PCa.

**Clinical trial registration:**

ClinicalTrials.gov, NCT05790213. Registered on March 30, 2023.

## Background

Prostate cancer remains a large public health burden, as it is the most common cancer diagnosis in men. Men with intermediate-risk (Grade Group (GG) ≥3) PCa represent approximately 48% of all PCa patients [1]. Many of these patients undergo whole gland treatments, which come with high morbidity [2]. The impact of these treatments on genitourinary and sexual function can result in devastating changes in quality of life.

In the last decade, focal therapy (FT) has emerged as a less invasive option for some men with biopsy proven clinically significant PCa (CSPCa) confined to a section of the prostate. FT is widely used, as a recent survey indicated almost half of US urologists utilize it in their practice [3]. Cryoablation, high-intensity focused ultrasound (HIFU), laser ablation, and irreversible electroporation are the more commonly used energy modalities for achieving FT. In contrast to whole gland treatment, FT considers the spatial distribution of PCa within the prostate for ablation, thus a fraction of tissue is destroyed to facilitate preservation of genitourinary function [4,5].

For decades surgeons have used prostate cryoablation as an alternative to surgical resection [6–8]. Cryoablation of the entire prostate has shown durable results for primary treatment and as salvage treatment for recurrent PCa. Studies of focal cryoablation indicate a lower rate of urinary incontinence and over 80% preservation of erectile function [9,10]. A systematic review of nine studies evaluating focal cryoablation from 2006–2014 by Shah et al. demonstrated a biochemical recurrence free survival percentage of 72% to 93% over the follow-up period of 9–70 months [11] 98 patients of the 291 that had post-procedural biopsies (25%) were found to have residual PCa, but ~68 specimens were identified as GG1 disease on the contralateral untreated portion of the prostate. Importantly, the percentage of urinary incontinence ranged from only 0–4% and erectile dysfunction ranged from 0 to 42%.

HIFU is another FDA-approved minimally-invasive FT modality used to treat low- to intermediate-risk PCa [12,13]. The mechanical energy of the focused ultrasound is converted into heat causing hyperthermia above 65°C leading to coagulative necrosis [14–16]. Stabile et al published one of the largest studies of patients receiving focal HIFU for mostly intermediate risk PCa [17]. The excellent oncological outcomes of focal HIFU was demonstrated by 91% of the men avoiding salvage whole gland treatments at five years. HIFU can also be used as a salvage therapy following biochemical recurrence after radiation therapy for PCa, or a first line salvage treatment combined with ADT for local recurrent PCa [18,19].

However, there are several limitations to FT. The location of the PCa lesion can affect treatment-related morbidity, for example, close proximity to the urethra and or rectum increases the risk for the rare complication of genitourinary fistula. There is yet to be a consensus with respect to the appropriateness of patient selection for PSA thresholds beyond 10 ng/mL. Long-term follow-up and outcomes of FT are currently under study and ultimately necessary to facilitate the creation of practice guidelines. Compliance with routine post treatment follow-up, including PSA, mpMRI and prostate biopsies are necessary and can affect patient selection, especially if there is concern a patient will be non-compliant [20].

Given a greater risk of local recurrence, the majority of patient selection for FT is reserved for low- or favorable intermediate-risk PCa [7,18]. Patients with large volume favorable intermediate risk or unfavorable intermediate-risk PCa are offered FT often solely in an investigational context due to the more aggressive nature of their disease and concern for local or distant treatment failure.

The dependence of prostate cancer on androgens for growth has been established for almost a hundred years [21]. Surgical castration is the simplest form of androgen deprivation therapy and is still used today. However, medications are now often used as a form of androgen deprivation therapy. Gonadotropin releasing hormone (GnRH) agonists, such as leuprolide, induce medical castration by downregulating GnRH receptors and after an initial surge, leading to decreased production of luteinizing hormone and follicle stimulating hormone, which results in a decreased release of androgens from the testicles [22]. ADT has long been used as a treatment to slow the progression of metastatic hormone-sensitive PCa [23,24]. However, ADT has also been shown to improve disease-free and overall survival for patients with locally advanced PCa when combined with RT [25]. The use of whole gland ablation combined with ADT for non-metastatic PCa has also previously been studied. Donnelly et al. randomized 244 patients with intermediate and high-risk PCa to 3 months of neoadjuvant ADT with RT or whole gland cryoablation [26]. They reported similar overall survival and biochemical recurrence free survival between the interventions and decreased PCa identified at surveillance 36-month prostate biopsy for patients who underwent ablation. Chin et al. randomized 64 men to 6-months of perioperative ADT and either RT (n = 31) or whole gland cryoablation (n = 33) and reported similar disease-specific and overall survival between the two treatments [27]. In order to utilize FT for higher risk classifications of PCa while obtaining radical treatment survival rates, a multimodal approach needs to be evaluated. Herein, our objective is to evaluate the safety and efficacy of combining ADT and FT to treat intermediate-risk PCa.

The primary objectives of the study include:

1. To determine the proportion of men with residual/recurrent clinically significant prostate cancer (Grade Group ≥2 disease) in the ablated or unablated prostate tissue following the combination treatment of 3-months of ADT and FT of the prostate in men with newly diagnosed non-metastatic intermediate risk prostate cancer; specifically, men with a histopathologic diagnosis of Grade Group 2 & 3, with prostate-specific antigen level <20 ng/mL.
2. To assess the safety of the combination treatment of ADT and FT of the prostate for the management of patients with newly diagnosed non-metastatic intermediate risk prostate cancer.

Secondary objectives include:

1. To assess the genitourinary side effect profile of the combination treatment of ADT and FT of the prostate as it pertains to lower urinary tract symptoms and sexual function based on health-related quality of life measures.
2. To determine the prostate specific antigen response following treatment with ADT and FT of the prostate.
3. To determine the proportion of men converting to whole gland therapy and/or requiring systemic therapy and/or developing metastases and/or dying of prostate cancer during the course of study.
4. To determine the proportion of men with absence of any cancer in the ablated or unablated prostate tissue on post treatment prostate biopsy.
5. To determine the proportion of men with baseline normal serum testosterone who have achieved testosterone recovery over the course of the study and follow-up period.

### Study design

**Fig 1:**
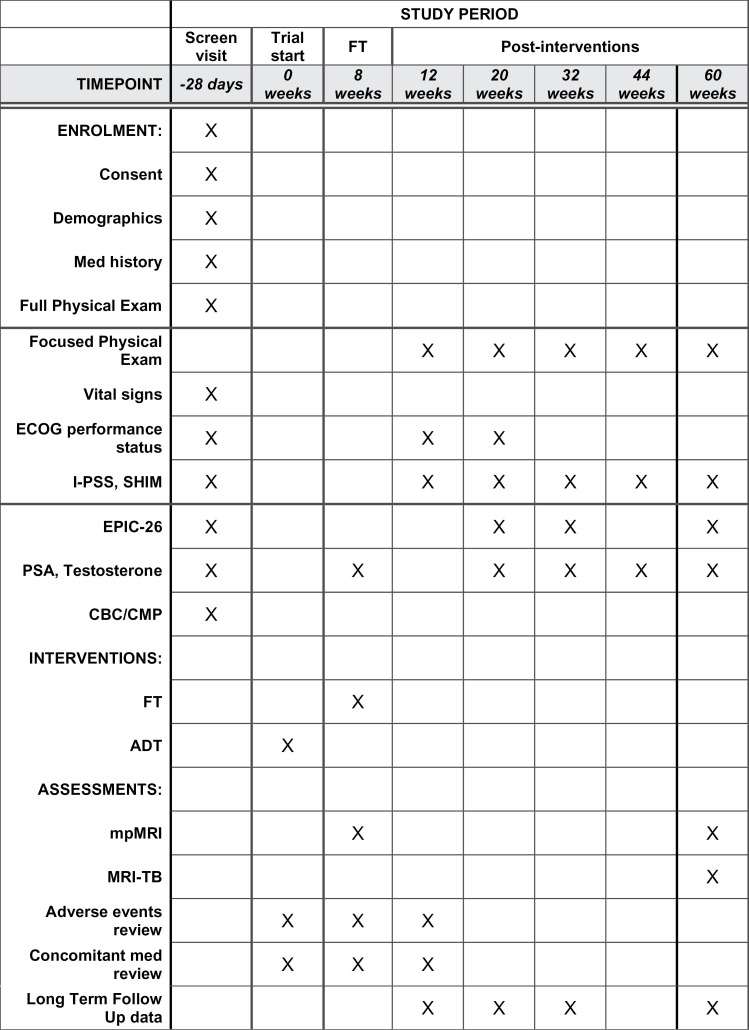
SPIRIT schedule. Enrollment, Intervention, and Assessment schedule. ECOG: Eastern Cooperative Oncology Group; I-PSS: International Prostate Symptom Score; SHIM: Sexual Health Inventory for Men; EPIC-26: Expanded Prostate Cancer Index Composite-26.

**Fig 2:**
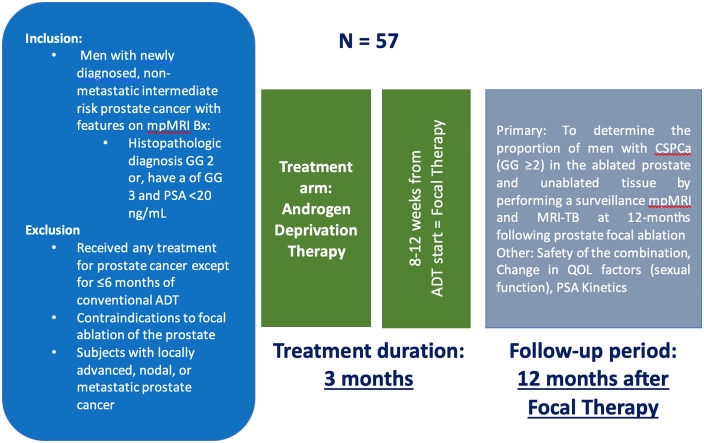
Study design overview.

The purpose of this phase II clinical trial is to determine the proportion of men with residual/recurrent clinically significant prostate cancer following the combination treatment of ADT and FT of the prostate in men with non-metastatic intermediate risk prostate cancer; defined as, men with a histopathologic diagnosis of Grade Group 2 & 3, with prostate specific antigen level <20 ng/mL. The study will open at the University of Chicago in 2025. IRB approval was obtained through the University of Chicago (IRB24–0259). The study is registered on ClinicalTrials.gov (NCT05790213). Recruitment began 05/05/2025 and expects to be completed by 07/01/2026. Completion of data collection is anticipated by 01/01/2028. The data and results from the study expect to be published 06/01/2028. The full study protocol and complete SPIRIT checklist can be found in the supporting information in S1 Checklist and S1 Protocol. Fig 1 outlines the timeline of the study design in the SPIRIT schedule. An overview of the study design is presented in Fig 2.

### Endpoints

The study endpoints are summarized in Table 1.

**Table 1:** Primary and Secondary Endpoints.

Primary endpoints
1. To determine the proportion of men with CSPCa (GG ≥ 2) in the ablated prostate tissue as evaluated by a surveillance mpMRI and MRI-TB at 12-months following FT.
2. To determine the proportion of men with CSPCa (GG ≥ 2) in the unablated prostate tissue as evaluated by a surveillance mpMRI and MRI-TB at 12-months following FT.
3. To demonstrate the safety of combining ADT and FT for the treatment of men with histopathologic diagnosis of GG 2 & 3 PCa, with PSA level < 20 ng/mL utilizing the NCI’s CTCAE v.5 classification to quantify and characterize the incidence of AEs.
Secondary endpoints
1. To define change in genitourinary and sexual function from baseline following ADT and FT by measuring the subject’s HRQoL (I-PSS, SHIM, EPIC-26) at 6-and 12-months after FT.
2. To determine the PSA response to the combination treatment by measuring the subject’s PSA at “baseline” (PSA at time of initial diagnosis) and then at 3-months, 6-months, and 1-year from FT.
3. To determine the proportion of men converting to whole gland therapy (radical prostatectomy or radiation therapy) and/or requiring systemic therapy and/or developing metastases and/or dying of PCa during the course of study.
4. To determine the proportion of men without any PCa on any post treatment prostate biopsy.
5. To determine the proportion of men with normal baseline serum testosterone who had testosterone recovery (defined as testosterone levels >300 ng/dL) at 6-, 9- and 12-months after FT.

### Eligibility criteria

The Inclusion and exclusion criteria for participants in the trial are listed in Table 2.

**Table 2:** Inclusion and Exclusion Criteria.

Inclusion criteria
1. Subjects must have intermediate-risk PCa as defined by the below criteria:
a. Favorable intermediate-risk PCa: i. ≤ clinical stage T2c, GG2, and PSA ≤ 10 ng/mL, and <50% positive biopsy cores with PCa
b. Unfavorable intermediate-risk PCa: i. ≤ clinical stageT2c, GG2, and PSA 10–20 ng/mL, or ≥50% positive biopsy cores with PCa, or ii. ≤ clinical stage T2c, GG3, and PSA < 20 ng/mL
2. No mpMRI evidence of extra-prostatic extension (EPE) or seminal vesicle invasion, and if seminal vesical invasion is suspected, it must be excluded by prostate biopsy.
3. Subjects must have chosen to get Focal Therapy for the treatment of prostate cancer.
4. Subjects must have confirmed non-metastatic PCa following standard of care (SOC) screening for patients with unfavorable intermediate-risk PCa, a combination of computed tomography imaging of the abdomen and pelvis (CTAP) and technetium-99-mDP nuclear medicine bone scan (BS) and/or prostate-specific membrane antigen positron emission tomography (PSMA/PET) scan prior to enrollment. The imaging studies should be obtained within 6-months of enrollment.
5. Subject must be male ≥ 18 years-old.
6. Subjects must have a life expectancy of at least 10-years per the opinion of the treating investigator.
7. Subjects must be designated as Eastern Cooperative Oncology Group (ECOG) performance status ≤ 2 or Karnofsky Performance Status Scale Score ≥ 60%, see Appendix A).
8. Subjects must be fit to undergo general anesthesia and the FT surgical procedure, which includes adequate visualization of the prostate gland on transrectal ultrasound imaging, access to the urethra, perineum and rectum, as well as be tolerant of lithotomy positioning in the opinion of the treating investigator or the operating surgeon(s) if not the same as the treating investigator.
9. Subjects with a prior or concurrent malignancy whose natural history or treatment does not have the potential to interfere with the safety or efficacy assessment of the investigational regimen are eligible for this trial.
10. Subjects who are sexually active with a woman of childbearing potential must agree to use a condom with spermicidal foam/gel/film/cream/suppository and his partner must also be practicing a highly effective method of contraception during treatment and for 3-months following the last ADT treatment.
11. Ability to understand and the willingness to sign a written informed consent document.
Exclusion criteria
1. Subject has had prior or current PCa therapies, such as biologic, chemotherapy, hormone therapy, radiotherapy or surgery for PCa. Subjects may not have had undergone pelvic radiation, chemotherapy or immunotherapy treatment for a separate hematologic or visceral malignancy within 6-months of enrollment in the present study.
2. Subjects with locally advanced, nodal or metastatic prostate cancer.
3. Subjects who are unfit for pelvic mpMRI scanning.
4. History of allergy or intolerance to study drug components.
5. History of bilateral orchiectomy.
6. If the subject has an uncontrolled or major debilitating inter-current illness.
7. Subjects who are receiving any other investigational agents, or who have received other investigational agents in the past and who are no longer receiving these investigational agents may be eligible at the discretion of the principal investigator (PI).
8. Judgment by the treating investigator or PI that the subject is unsuitable to participate in the study and the subject is unlikely to comply with study procedures, restrictions, and requirements.

## Methods

### Androgen deprivation therapy

Subjects will initiate ADT via any generic luteinizing hormone-releasing hormone agonist such as leuprolide acetate 22.5 mg subcutaneous 3-month injection once at baseline for a total of 3-months of treatment. ADT will be dosed to complete 3-months of pharmacologic treatment per study protocol. Of note, each participant will complete at least 8-weeks of neoadjuvant treatment prior to FT.

### Imaging

After pharmacological treatment has been started, there is an anticipated decrease in prostate volume. A change in prostate volume can change the specific spatial location of a prostate lesion, affecting FT planning. To identify if any significant shifts in the size and or location of the prostate lesion(s) have occurred, mpMRI will be performed eight weeks after initiation of pharmacological treatments and before FT. This mpMRI will be compared against the initial diagnostic mpMRI imaging. All mpMRI imaging will utilize the SOC Prostate Imaging Reporting and Data System Version 2.1 (PI-RADS). Radiologists with expertise in pelvic mpMRI will assign a PI-RADS score to each lesion(s) and identify the specified distribution of the lesion(s).

### Focal therapy treatment

The FT procedure used in this study does not differ from FT in clinical care and is not altered for research purposes. FT will be performed 8 weeks after initiation of ADT. Both mpMRIs and the discretion of the surgeon(s) are necessary to determine the volume and precise location(s) of prostate tissue to be treated. Only FT via cryoablation or HIFU will be performed. The choice of modality will depend on the location of targeted treatment (posterior lesions for HIFU, cryoablation can be used in all locations), patient preferences, and the surgeon(s) discretion.

The FT procedure will be performed using the target(s) identified from the subject’s screening MRI-TB. For larger template ablation or for cognitive targeting (operator ablates prostate tissue seen on live ultrasound that he/she recalls as the location of a lesion on the subject’s mpMRI should the operator choose to perform the procedure in this specific fashion), the standard FT equipment without fusion of mpMRI and prostate ultrasound imaging will be used.

The ablation template for FT must adhere to the following principles:

1. A maximum of 60–75% prostate ablation to include all CSPCa.
2. Treatment may reach the urethra and may cross the midline anteriorly or posteriorly.
3. At least one neurovascular bundle must be avoided by ensuring a minimum distance of ablation zone border to the contralateral neurovascular bundle of 5 mm. If this is not possible, the patient will not be considered for FT.
4. Up to one redo FT treatment is allowed; if either the protocol’s 6-month MRI-TB result(s) of ablated prostate tissue, or a ‘for-cause’ prostate biopsy (see “for cause tests”) are positive during follow-up.

### Efficacy & surveillance

Twelve months after FT, the efficacy of the combination treatment will be evaluated with a mpMRI and MRI-TB. The MRI-TB will sample the prostate tissue to determine if residual CSPCa (GG ≥ 2) remains in the treated and/or untreated prostate tissue. The mpMRI used for the MRI-TB will follow the same protocol as the pre-treatment imaging including documentation of the biopsy site(s). If a suspicious radiographic lesion(s) is/are detected on this mpMRI which was/were not present in the screening mpMRI then targeted biopsy cores of the suspicious area(s) will be obtained.

If CSPCa is identified in the 12-month MRI-TB then the patient will be offered further care outside of the clinical trial.

### PSA and testosterone testing

A “baseline” PSA value must be obtained before the MRI-TB that provided the initial histopathologic diagnosis. A “baseline” serum testosterone level must be obtained at screening visit unless there is already a serum testosterone level in the last three months.

Additionally, PSA and serum testosterone testing results will be collected at 8-weeks after initiation of neoadjuvant therapy and at 3,6,9 and 12-month clinic visits following FT per SOC. SOC intervals will be used for patient follow up after the 12-month visit. If a subject shows an increase in PSA (>2 ng/mL higher than baseline PSA) at 8 weeks after initiation of neoadjuvant therapy, he will be considered to have biochemical progression and will be withdrawn from the study. He will be offered clinical care based on re-staging work-up per SOC.

### For cause tests

If there is a significant increase in PSA levels (≥2 ng/mL higher from the subject’s post-FT PSA nadir, i.e., ‘for cause’ prostate biopsy) or clinical suspicion for progression of PCa clinicians can choose to proceed with prostate biopsies. If prostate inflammation/infection is suspected due to a biopsy or urinary tract infection, patients will undergo a course of antibiotics before repeating PSA. If the PSA again demonstrates a rise of 2 ng/mL above nadir, the patient will be evaluated for local disease recurrence. The specimens from a biopsy will be processed according to standard protocol and examined by a pathologist to note for every core the presence of adenocarcinoma, grade group, and length of cancer and total core length.

### Health-related quality of life questionnaires

The following questionnaires will be used to assess the patient’s health-related quality of life (HRQoL), and the answers will be selected at the screening visit and at follow-up visits. The International Prostate Symptom Score (I-PSS) the I-PSS provides a measure of urinary symptoms of benign prostate hyperplasia. Sexual Health Inventory for Men (SHIM) potency score assesses erectile function. Expanded Prostate cancer Index Composite Short Form (EPIC-26) is a subject-validated PCa HRQoL instrument that assesses a wide variety of urinary, bowel, sexual, and hormonal symptoms.

### Sample size

It is estimated that 30% of men who undergo FT for intermediate-risk PCa will have CSPCa (GG ≥ 2) on repeat biopsy after FT. We hypothesize the combination treatment will halve the proportion of men with residual or recurrent CSPCa to 15%. Using sample size estimates for a single proportion, comparing a null proportion of 0.30 to an alternative proportion of 0.15, with an alpha = 0.05 and power of at least 80%, the total number of patients required will be 51 men. We used one-sided proportion Z test for sample size calculation. We estimate 10% attrition for a total sample size of 57 men.

### Analysis of primary and secondary endpoints

All statistical analyses will be performed using SAS software (V9.4, SAS Institute Inc., Cary, NC). Analysis will be conducted via an intent-to-treat (ITT) population and a per-protocol (PP) population. The ITT population includes all subjects who were enrolled and treated with the combination treatment (ADT (any duration) + FT). The ITT population will serve as the primary analysis set for all safety and efficacy endpoints. The PP population includes all subjects who were enrolled and treated with the combination treatment (ADT (any duration) + FT), and who had no major protocol deviations. The PP population will serve as a supportive analysis set.

In the case of missing data, the following imputation methods will be used for the primary analysis of all endpoints:

- When calculating the proportion of subjects with a negative biopsy for CSPCa, subjects with missing biopsy information post-ablation will be imputed as “positive”
- Missing PSA data will be imputed using last observation carried forward (or previous median/mean or regression-based methods if proper).
- Missing data for IIEF-5, IPSS and EPIC questionnaires will be imputed using a multiple imputation approach at the item/question level.

All other endpoints/data (e.g., safety events) will be analyzed based on available (non-imputed) data, unless specified otherwise.

The primary objective will be expressed as proportions of total number of patients and the 95% confidence interval of the proportion will be presented. Number and grade of complications will be reported. HrQOL measures collected at 6 and 12 months will be compared to baseline measures using paired tests. In addition, the 6-month and 12-month change from baseline will also be tabulated.

The mean PSA levels at each visit, the overall percent reduction in PSA levels from baseline to each visit, and the proportion of subjects with a PSA reduction compared to baseline at each visit will be presented. Mean PSA nadir and post-nadir PSA values through 12-months post-treatment will be summarized. The number of men who have recovered normal serum testosterone levels will be expressed as proportions of total number of eugonadal patients and the 95% confidence interval of the proportion will be presented. The number and proportion of subjects undergoing secondary treatment during the study will be presented.

## Discussion

The high morbidity and mortality associated with a diagnosis of PCa necessitate more options for treatment. Particularly, more successful treatment outside of whole gland interventions, due to the high treatment related morbidity [2]. Recently, FT has become a treatment option for lower risk PCa with less side effects than whole gland approaches [3]. However, FT leads to a higher risk of local recurrence of PCa compared to whole gland therapies.^11^ In order to increase the efficacy of FT, while attempting to maintain a favorable adverse effect profile, the combination of FT with another therapy deserves investigation.

ADT is a well-established treatment modality for PCa. Additionally, ADT is already being used in many combination treatments, and it is increasingly showing efficacy as part of new combination treatments [28,29]. A recent phase II trial indicated that stereotactic body radiotherapy has greater progression free survival when combined with a short course of ADT for oligorecurrent PCa [30]. This study seeks to evaluate if the efficacy of FT will be augmented by the addition of ADT, similar to the success obtained by other combination treatments with ADT. Specifically this phase II clinical trial seeks to evaluate local recurrence rates of PCa after combining FT with ADT in patients with intermediate risk localized PCa. The safety of this multimodal approach to PCa treatment will also be evaluated.

This is the first phase II trial of its kind comparing the combination of FT with ADT for the primary treatment of PCa. If successful, this approach could offer patients a new option for treatment with fewer long-term side effects than whole gland interventions and more efficacy than FT alone. Additionally, the success of this trial could further increase the popularity of FT, as clinicians may be more likely to utilize a novel treatment, such as FT, when it is combined with a traditional therapy, such as ADT. More research is still needed to evaluate other options for therapies in combination with FT for PCa, and the results of this study may inspire other studies of effective and safe multimodal treatment options for patients diagnosed with PCa.

## Supporting information

S1 Checklist The SPIRIT checklist. (DOC)

S1 Protocol The full trial protocol. (DOCX)
