# Supplementary material for: Phase II trial protocol of focal prostate ablation combined with androgen deprivation therapy for prostate cancer treatment
Source: PLoS One. 2025 Dec 16;20(12):e0337828. doi: 10.1371/journal.pone.0337828 (PMC12707638; doi:10.1371/journal.pone.0337828)
Supplement: S1 Protocol — (DOCX) [file pone.0337828.s002.docx]

**University of Chicago Protocol #:** IRB24-0259

**TITLE:** Phase II Trial of Focal Prostate Ablation Combined with Androgen Deprivation Therapy for Prostate Cancer Treatment

**Coordinating Center:** *University of Chicago Medicine Comprehensive Cancer Center*

*(UCCCC)*

**Principal Investigator:** Abhinav Sidana, MD, MPH

The University of Chicago

5841 S. Maryland Ave.. MC 2115

Chicago, IL 60637

abhinav.sidana@bsd.uchicago.edu

*This study is being conducted by the Clinical Trials Support Office at the University of Chicago Comprehensive Cancer Center, as well as additional sites.*

**Protocol Version Date:** October 10, 2024

**PROTOCOL HISTORY**

*Use the table below to summarize modifications and amendments to protocol that have been approved by Dr. Sidana (Principal Investigator).*

| **Protocol Version Date** | **Protocol Type** *(Original, Amendment)* |
| --- | --- |
| **v1.30.2024** | **Original Protocol** |
| **v10.4.2024** | **Revised Protocol** |

# SCHEMA

**Inclusion:**

- Men with newly diagnosed, non-metastatic intermediate risk prostate cancer with features on mpMRI Bx:
  - Histopathologic diagnosis GG 2 or, have a of GG 3 and PSA <20 ng/mL

**Exclusion**

- Received any treatment for prostate cancer except for ≤6 months of conventional ADT
- Contraindications to focal ablation of the prostate
- Subjects with locally advanced, nodal, or metastatic prostate cancer

**Treatment arm: Androgen Deprivation Therapy**

8-12 weeks from

ADT start = Focal Therapy

Primary: To determine the proportion of men with CSPCa (GG ≥2) in the ablated prostate and unablated tissue by performing a surveillance mpMRI and MRI-TB at 12-months following prostate focal ablation

Other: Safety of the combination, Change in QOL factors (sexual function), PSA Kinetics

**N = 57**

**Treatment duration: 3 months**

**Follow-up period: 12 months after Focal Therapy**

# STUDY ABBREVIATIONS

The following are common terms and abbreviations and acronyms used throughout this protocol. The terms “focal therapy,” “focal prostate ablation,” and “prostate ablation” are considered to be interchangeable for purpose of this trial.

| **Abbreviation** | **Term/Description** |
| --- | --- |
| **ADT** | Androgen Deprivation Therapy – any generic LHRH |
| **AE** | Adverse Event |
| **AR** | Androgen Receptor |
| **BS** | Bone Scan |
| **CBC** | Complete Blood Count |
| **CMP** | Complete Metabolic Panel |
| **ConMed** | Concomitant medication |
| **CS / NCS** | Clinically significant / Not clinically significant |
| **CSPCa** | Clinically significant prostate cancer |
| **CTAP** | Computed tomography arterial portography |
| **CTCAE** | Common Terminology Criteria for Adverse Events |
| **ECOG** | Eastern Cooperative Oncology Group, a Performance Scale |
| **EDC** | Electronic Data Capture – REDCap system in use for this study |
| **EOT** | End of Treatment |
| **EPE** | Extraprostatic extension |
| **EPIC-26** | Expanded Prostate cancer Index Composite short form questionnaire |
| **FT** | Focal Therapy ablation of the prostate |
| **GG** | Grade Group and not “Gleason grade” which refers to the older system of Gleason sum (6-10) |
| **HIFU** | High-Intensity Focused Ultrasound |
| **HRQoL** | Health related quality of life |
| **I-PSS** | International prostate symptom score |
| **LHRH** | Luteinizing hormone-releasing hormone |
| **LTFU** | Long term follow-up |
| **mpMRI** | Multiparametric magnetic resonance imaging |
| **MRI-TB** | Magnetic resonance imaging targeted biopsy |
| **PCa** | Prostate cancer |
| **PFS** | Progression free survival |
| **PI** | Principal investigator |
| **PI-RADS** | Prostate Imaging Reporting & Data System |
| **PSA** | Prostate Specific Antigen |
| **PE** | Physical Exam |
| **RT** | Radiation Therapy |
| **SAE** | Serious Adverse Event |
| **SHIM** | Sexual Health Inventory for Men |
| **SOC** | Standard of Care – per institution |
| **TPM** | Template mapping biopsies |
| **UA** | Urinalysis |
| **CTSO** | Clinical Trial Support Office |
| **UCCCC** | University of Chicago Medicine Comprehensive Cancer Center |

**TABLE OF CONTENTS**

[1. SCHEMA 3](#_Toc177730194)

[2. STUDY ABBREVIATIONS 4](#_Toc177730195)

[3. OBJECTIVES 8](#_Toc177730196)

[3.1. Primary Objectives 8](#_Toc177730197)

[3.2. Secondary Objectives 8](#_Toc177730198)

[3.3. Exploratory Objectives 8](#_Toc177730199)

[4. BACKGROUND 8](#_Toc177730200)

[4.1. Prostate Cancer Background 8](#_Toc177730201)

[4.2. Prostate Cryoablation and High-Intensity Focused Ultrasound Ablation for Focal Therapy Treatment of Prostate Cancer 9](#_Toc177730202)

[4.3. Rationale for the Combination of Focal Therapy with Androgen Deprivation Therapy 10](#_Toc177730203)

[4.4. Correlative Studies Background 11](#_Toc177730204)

[5. PATIENT SELECTION: ELIGIBILITY 11](#_Toc177730205)

[5.1. Inclusion Criteria 11](#_Toc177730206)

[5.2. Exclusion Criteria 13](#_Toc177730207)

[5.3. Inclusion of Women and Minorities 14](#_Toc177730208)

[6. SCREENING & REGISTRATION PROCEDURES 14](#_Toc177730209)

[6.1. General Guidelines 14](#_Toc177730210)

[6.2. Registration Procedures 14](#_Toc177730211)

[6.3. Assignment of Screening and Subject Study Numbers 15](#_Toc177730212)

[6.4. Screen Failures & Re-Screening 15](#_Toc177730213)

[6.5. Subjects who do not undergo Focal Therapy or complete Focal Therapy 16](#_Toc177730214)

[6.6. Screening Prostate Cancer Localization by Multiparametric MRI 16](#_Toc177730215)

[6.7. Screening Prostate Cancer Diagnosis by Systematic and MRI Targeted Prostate Biopsy 16](#_Toc177730216)

[6.8. Standard of Care Imaging for Eligibility 17](#_Toc177730217)

[6.9. Timeframes for Screening Procedures 17](#_Toc177730218)

[7. BIOMARKER, CORRELATIVE, AND SPECIAL STUDIES 17](#_Toc177730219)

[7.1. Biomarker Plan 17](#_Toc177730220)

[7.2. Correlative Studies 18](#_Toc177730221)

[7.3. Exploratory/Ancillary Correlative Studies 18](#_Toc177730222)

[7.4. Peripheral Blood or Tissue 18](#_Toc177730223)

[8. TREATMENT PLAN 19](#_Toc177730224)

[8.1. Health-related Quality of Life Assessments 19](#_Toc177730225)

[8.2. Laboratory Testing 20](#_Toc177730226)

[8.3. Pharmacological Research Treatments: Androgen Deprivation Therapy 20](#_Toc177730227)

[8.4. Focal Therapy Procedure Combination with Androgen Deprivation therapy 21](#_Toc177730228)

[8.5. Post-Focal Therapy Treatment Efficacy/Surveillance mpMRI and MRI-TB 27](#_Toc177730229)

[8.6. For Cause Tests 27](#_Toc177730230)

[9. TOXICITY MANAGEMENT GUIDELINES/SAFETY MONITORING 28](#_Toc177730231)

[9.1. Hypothyroidism 28](#_Toc177730232)

[9.2. Falls and fractures 28](#_Toc177730233)

[9.3. Ischemic heart disease and ischemic cerebrovascular disorders 28](#_Toc177730234)

[9.4. General Concomitant Medication and Supportive Care Guidelines 29](#_Toc177730235)

[10. DURATION OF TREATMENT, FOLLOW-UP & WITHDRAWALS 29](#_Toc177730236)

[10.1. Duration of Therapy 29](#_Toc177730237)

[10.2. Duration of Follow-Up 29](#_Toc177730238)

[10.3. Lost to follow-up 30](#_Toc177730239)

[10.4. Withdrawal of Consent 30](#_Toc177730240)

[11. OTHER RESEARCH ACTIVITY SPECIFICATIONS 30](#_Toc177730241)

[11.1. Medical History 31](#_Toc177730242)

[11.2. Prior and Concomitant Medications 31](#_Toc177730243)

[11.3. Adverse Events 31](#_Toc177730244)

[11.4. Full Physical Exam 31](#_Toc177730245)

[11.5. Focused Physical Exam 31](#_Toc177730246)

[11.6. Vital Signs 32](#_Toc177730247)

[11.7. Eastern Cooperative Oncology Group (ECOG) Performance Scale 32](#_Toc177730248)

[11.8. Assessment of Disease 32](#_Toc177730249)

[11.9. Laboratory Safety Evaluations (Hematology, Chemistry and Urinalysis) 32](#_Toc177730250)

[12. PHARMACEUTICAL INFORMATION 32](#_Toc177730251)

[12.1. Investigator Brochure Availability 33](#_Toc177730252)

[13. STATISTICAL CONSIDERATIONS 33](#_Toc177730253)

[13.1. Study Design/Endpoints 33](#_Toc177730254)

[13.4. Sample Size/Accrual Rate 34](#_Toc177730255)

[13.5. Stratification Factors 34](#_Toc177730256)

[13.6. Analysis of Primary and Secondary Endpoints 34](#_Toc177730257)

[14. SAFETY EVENT AND PRODUCT QUALITY COMPLAINTS REPORTING – PRINCIPAL INVESTIGATOR RESPONSIBILITIES 37](#_Toc177730258)

[14.1. Health Authority 37](#_Toc177730259)

[General 37](#_Toc177730260)

[14.2. 37](#_Toc177730264)

[14.3. Adverse Event (AE) Definitions 37](#_Toc177730265)

[14.4. Serious Adverse Event Definitions 38](#_Toc177730266)

[Any adverse event occurring that: 38](#_Toc177730267)

[14.5. Adverse Event Reporting Requirements 40](#_Toc177730268)

[14.6.1 Routine Adverse Events Reporting 40](#_Toc177730269)

[14 STUDY CALENDAR 42](#_Toc177730270)

[15 MEASUREMENT OF EFFECT 43](#_Toc177730271)

[16 STUDY MANAGEMENT AND REGULATORY AFFAIRS 43](#_Toc177730272)

[16.1 Multicenter Guidelines 43](#_Toc177730273)

[16.2 Institutional Review Board (IRB) Approval and Consent 43](#_Toc177730274)

[16.3 Required Documentation 44](#_Toc177730276)

[16.4 Trial Monitoring 44](#_Toc177730277)

[16.5 Auditing 45](#_Toc177730278)

[16.6 Amendments to the Protocol 45](#_Toc177730279)

[16.7 Annual IRB Renewals, Continuing Review and Final Reports 46](#_Toc177730280)

[16.8 Record Retention 46](#_Toc177730281)

[16.9 Obligations of Study Site Investigators 46](#_Toc177730282)

[17 REFERENCES 47](#_Toc177730283)

[18 APPENDIX A PERFORMANCE STATUS CRITERIA 49](#_Toc177730284)

[19 APPENDIX B Prostate Multiparametric Magnetic Resonance Imaging and Transperineal or Transrectal Magnetic Resonance Imaging Targeted Prostate Biopsy Protocols 50](#_Toc177730285)

[20 APPENDIX C: Quality of Life I-PSS 54](#_Toc177730286)

[21 APPENDIX D: Quality of Life – SHIM 55](#_Toc177730287)

[22 APPENDIX E: Quality of Life – EPIC-26 56](#_Toc177730288)

[24 APPENDIX F MULTICENTER GUIDELINES 56](#_Toc177730289)

# OBJECTIVES

## Primary Objectives

1. To determine the proportion of men with residual/recurrent clinically significant prostate cancer (Grade Group ≥2 disease) in the ablated or unablated prostate tissue following the combination treatment of 3-months of androgen deprivation therapy, and partial ablation of the prostate in men with newly diagnosed non-metastatic intermediate risk prostate cancer; specifically, men with a histopathologic diagnosis of Grade Group 2 & 3, with prostate specific antigen level <20 ng/mL.
2. To assess the safety of the combination treatment of androgen deprivation therapy and partial ablation of the prostate for the management of patients with newly diagnosed non-metastatic intermediate risk prostate cancer; specifically, men with a histopathologic diagnosis of Grade Group 2 & 3, with prostate specific antigen level <20 ng/mL using the common terminology criteria for adverse events.

## Secondary Objectives

1. To assess the genitourinary side effect profile of the combination treatment of androgen deprivation therapy, and partial ablation of the prostate as it pertains to lower urinary tract symptoms and sexual function based on health-related quality of life measures.

1. To determine the prostate specific antigen response following treatment with androgen deprivation therapy, and partial ablation of the prostate.
2. To determine the proportion of men converting to whole gland therapy (radical prostatectomy or radiation therapy) and/or requiring systemic therapy and/or developing metastases and/or dying of prostate cancer during the course of study.
3. To determine the proportion of men with absence of any cancer in the ablated or unablated prostate tissue on post treatment prostate biopsy.
4. To determine the proportion of men with baseline normal serum testosterone who have achieved testosterone recovery over the course of the study and follow-up period.

## Exploratory Objectives

1. To prospectively obtain tissue and blood for biobanking for use in future potential analysis or research on prostate cancer.

# BACKGROUND

## Prostate Cancer Background

The burden of prostate cancer (PCa) remains significant, with estimates of approximately 248,530 new diagnoses and 34,130 deaths from PCa in 2021.^1^ It is estimated that men with intermediate-risk and high-risk disease (Grade Group (GG) ≥3) represent 37-39% and 26-28% of all PCa patients, respectively.^2^ A majority of these patients undergo whole gland intervention, such as radiation therapy (RT) with androgen deprivation therapy (ADT) or radical prostatectomy, which results in significant treatment related morbidity.^3^ The impact of said treatments on genitourinary and sexual function can result in devastating changes in quality of life. Furthermore, a significant proportion of these patients can have yet unidentified micro-metastatic lesions that may progress to clinically or radiologically confirmed disseminated disease; thus, the risk of morbidities of a primary radical whole gland intervention may outweigh the benefit attained.

In the past decade, partial ablation of the prostate, or focal therapy (FT), has become a feasible treatment option for select men with biopsy proven clinically significant PCa (CSPCa) confined to a portion of the prostate. FT is delivered via a variety of modalities including cryoablation, high-intensity focused ultrasound (HIFU), and irreversible electroporation. In contrast to whole gland treatment, FT considers the spatial distribution of PCa within the prostate for ablation, thus a fraction of tissue is destroyed to facilitate preservation of genitourinary function.^4^

## Prostate Cryoablation and High-Intensity Focused Ultrasound Ablation for Focal Therapy Treatment of Prostate Cancer

Cryoablation, or tissue destruction by freezing, has been used as an alternative to surgical resection for decades.^5-8^ Cryoablation of the entire prostate has shown durable results for primary treatment and utilized as salvage treatment after primary RT for PCa.^9^ Studies of FT cryoablation have demonstrated a reduced rate of urinary incontinence and >80% preservation of erectile function.^10^ Oishi et al.^11^ prospectively evaluated 160 men who underwent cryoablation of localized PCa for oncologic and functional outcomes. Their primary endpoint was treatment failure-free survival, which was defined as requiring any radical and or whole gland treatment, initiation of systemic therapy, biochemical failure (increase of 2 ng/mL above the PSA nadir) or PCa specific mortality. Twenty-five men (16%) had high-risk PCa, 106 men (66%) had intermediate-risk and 29 men (18%) had low-risk PCa with an overall median baseline PSA of 6.3 ng/mL and follow-up of 40-months. Of note, 28 patients (17 intermediate-risk and 11 high-risk) received neoadjuvant ADT prior to FT for a median of 12-months. Transrectal ultrasound guided prostate biopsy was recommended at 1-year after FT cryoablation; however, 104 men ultimately underwent post-procedural prostate biopsy. The authors estimated that 5-year treatment failure-free survival, metastasis-free survival, cancer specific and overall survival were 85%, 100%, 100% and 100%, respectively. The 5-year CSPCa, i.e., GG ≥2, free survival was 87%, 74% and 64% for low-, intermediate- and high-risk subjects who underwent a post-procedural biopsy, respectively. Continence and potency were reported in 97% and 73% of patients, respectively, within 2-years of FT.

Shah et al.^12^ performed a systematic review of literature from 2006-2014 regarding FT cryoablation treatment of PCa. They included 9 studies with 1582 patients that underwent FT. All studies were case series with no control group. A sub-group analysis of 759 patients (48%) had intermediate- (~80%) or high-risk (~20%) disease based on transrectal or transperineal prostate biopsy with follow-up ranging from 9- to 70-months. The authors reported a rate of biochemical recurrence free survival of 72 to 93% over the follow-up period based on an increase of 2 ng/mL above nadir PSA or three consecutive increases in PSA from nadir following FT. Of the 291 patients that had post-procedural biopsy, 98 men (25%) were found to have residual PCa, but ~68 specimens were identified as GG1disease on the contralateral untreated portion of the prostate. Patient report of incontinence ranged from 0 to 4% and erectile dysfunction ranged from 0 to 42%.

Tay et al.^13^ provided recommendations for patient management based on a consensus panel of 47 international experts in FT. The panel endorsed the treatment of GG1, GG2 and GG3 disease using FT and provided tumor volume thresholds as guidance for patient selection. In addition, multiparametric magnetic resonance imaging (mpMRI) and combined systematic and mpMRI targeted prostate biopsy (MRI-TB) were deemed standard imaging and diagnostic testing necessary for FT candidacy. Lebastchi et al.^14^ assimilated an international expert panel and agreed that mpMRI is the preferred imaging modality for identifying the treatment response of FT, provided a definition and recommendations for patient follow-up, and suggested that MRI-TB within 1-year of FT to evaluate the treated and untreated prostate tissue should be standard practice.

HIFU is an FDA approved minimally-invasive procedure used as a targeted approach to treat low- to intermediate-risk PCa.^15, 16^ The focused ultrasound energy delivered to the prostate results in mechanical and thermal injury and cavitation of the tissue. The mechanical energy is converted into heat causing hyperthermia above 65°C leading to coagulative necrosis.^17-19^ HIFU can be used for partial as well as whole gland treatment of PCa and is currently the most commonly used energy modality for focal therapy of prostate cancer in US due to excellent functional outcomes in terms of erections and continence.Click or tap here to enter text.^20^ Stabile et al reported one of the largest experience of HIFU used for focal treatment of prostate cancer in 2019. Majority of their patients receiving focal HIFU had intermediate risk prostate cancer with only a small proportion having low risk prostate cancer. While not everyone got a repeat biopsy, 25% of the total population was found to have CS cancer on repeat biopsy. However, 91% of the men avoided radical therapy at 5 years highlighting the excellent oncological outcomes of focal HIFU in short and intermediate term. HIFU is also used as a salvage therapy following biochemical recurrence after radiation therapy for PCa.Click or tap here to enter text.^21^ HIFU has also been used along with ADT as first line salvage therapy for local recurrent Pca.^22, 23^Click or tap here to enter text.

There are limitations to FT.^24^ The location of the PCa lesion can affect treatment related morbidity, e.g., its proximity to the urethra and or rectum risks the rare complication of genitourinary fistula. There is yet undetermined consensus with respect to appropriateness of patient selection for PSA thresholds beyond 10 ng/mL. Long-term follow-up and outcomes of FT are currently under study and ultimately necessary to facilitate creation of practice guidelines. Compliance with routine and long-term follow-up, including repeat prostate biopsy following FT, is necessary and can affect patient selection, especially if there is concern a patient will be non-compliant. The appropriateness of FT may be limited to patient performance status and life expectancy, with expert opinion suggesting intermediate-risk PCa treatment guidelines be followed in this regard.

## Rationale for the Combination of Focal Therapy with Androgen Deprivation Therapy

Given a greater risk of local recurrence^17^, the majority of patient selection for FT is reserved for low- or favorable intermediate-risk PCa.^10^ Patients with unfavorable intermediate-risk PCa are offered FT often in the context of experimentation due to the more aggressive nature of their disease and concern for local or distant treatment failure.

The use of whole gland ablation for non-metastatic PCa in combination with ADT has previously been studied. Donnelly et al.^25^ randomized 244 patients with intermediate- and high-risk PCa to 3-months of neoadjuvant ADT with RT or whole gland cryoablation and reported similar overall and biochemical recurrence free survival between interventions and decreased PCa identified at surveillance 36-month prostate biopsy for patients who underwent ablation. Chin et al.^26^ randomized 64 men to 6-months of perioperative ADT and RT (n=31) or whole gland cryoablation (n=33) and reported similar disease-specific and overall survival between the two treatments. Thus, efforts to manage intermediate-risk or greater risk classifications of PCa utilizing FT would require a multimodal approach and could lead to similar survival rates as radical treatment.

We believe that the effective androgen blockade will augment the oncologic success of FT. Herein, our objective is to investigate the safety and efficacy of combining ADT and partial ablation of the prostate for the treatment of intermediate -risk PCa.

## Correlative Studies Background

Given this novel combination therapy, we wish to obtain core tissue samples at the time of FT for biospecimen banking and potential future analysis and research on PCa. Two 18-gauge biopsy cores will be collected from the target area(s) and one each from peritumoral and untreated prostate for exploratory outcomes on correlatives during the FT procedure.

At the time of 12-month MRI-TB two 18-gauge biopsy cores will be collected from the ablated area(s) and one each from periablation zone and untreated prostate for exploratory outcomes on correlatives during the FT procedure.

# PATIENT SELECTION: ELIGIBILITY

## Inclusion Criteria

1. Subjects must have intermediate-risk PCa as defined by the below criteria:
   1. Favorable intermediate-risk PCa:
      1. ≤ clinical stage T2c, GG2, and PSA ≤ 10 ng/mL, and <50% positive biopsy cores with PCa
   2. Unfavorable intermediate-risk PCa:
      1. ≤ clinical stageT2c, GG2, and PSA 10-20 ng/mL, or ≥50% positive biopsy cores with PCa, or
      2. ≤ clinical stage T2c, GG3, and PSA < 20 ng/mL

Note: The PSA value for this inclusion criteria must be the value obtained just prior to the subject’s MRI-TB that provided the initial histopathologic diagnosis. This is considered to be the subject’s “baseline” PSA.

If the MRI-TB which initially diagnosed the subject’s PCa was obtained greater than 3-months from the time of study consent, then a repeat PSA should be completed for screening purposes to obtain a “baseline” PSA (unless one has been obtained for SOC at least 3-months after this initial biopsy in which case no repeat value is needed and this may be used for eligibility). This applies to all participants regardless of GG used for eligibility.

Note: The histopathologic diagnosis must be obtained via “MRI-TB”, which for the purposes of the present study, is defined as both a systematic 12-core sextant random prostate biopsy and a targeted prostate biopsy. The targeted prostate biopsy can be performed via in-bore mpMRI prostate biopsy, cognitive mpMRI/ultrasound fusion prostate biopsy or software mpMRI/ultrasound fusion prostate biopsy. This “MRI-TB” must not be obtained greater than 1 year from the date of consent. See section 6.6. for more requirements for the MRI-TB.

1. No mpMRI evidence of extra-prostatic extension (EPE) or seminal vesicle invasion, and if seminal vesical invasion is suspected, it must be excluded by prostate biopsy.
2. Subjects must have chosen to get Focal Therapy for the treatment of prostate cancer.
3. Subjects must have confirmed non-metastatic PCa following SOC screening for patients with unfavorable intermediate-risk PCa, a combination of computed tomography imaging of the abdomen and pelvis (CTAP) and technetium-99-mDP nuclear medicine bone scan (BS) and/or prostate-specific membrane antigen positron emission tomography (PSMA/PET) scan prior to enrollment. The imaging studies should be obtained within 6-months of enrollment. Additional imaging is not required for men with favorable intermediate-risk PCa. See [Section 6.7](#_Screening_Prostate_Cancer).
4. Subject must be male ≥ 18 years-old.
5. Subjects must have a life expectancy of at least 10-years per the opinion of the treating investigator.
6. Subjects must be designated as Eastern Cooperative Oncology Group (ECOG) performance status ≤ 2 or Karnofsky Performance Status Scale Score ≥ 60%, see Appendix A).
7. Subjects must be fit to undergo general anesthesia and the FT surgical procedure, which includes adequate visualization of the prostate gland on transrectal ultrasound imaging, access to the urethra, perineum and rectum, as well as be tolerant of lithotomy positioning in the opinion of the treating investigator or the operating surgeon(s) if not the same as the treating investigator.
8. Subjects must have adequate organ and marrow function as defined below:

| Hemoglobin | ≥ 10 g/dL |
| --- | --- |
| Leukocytes | ≥ 3,000/mcL |
| Absolute neutrophil count | ≥ 1,500/mcL |
| Platelets | ≥ 100,000/mcL |
| Total bilirubin | ≤ 1.5 x institutional upper limit of normal (ULN) |
| AST(SGOT)/ALT(SGPT) | ≤ 2.5 × institutional ULN |
| Creatinine | < 1.5 institutional ULN |
| OR |  |
| Calculated or measured creatinine clearance | > 50 mL/min/1.73 m^2^ |
| eGFR | >30 mL/min using the MDRD (modification of diet and renal disease) formula |
| Serum albumin | ≥3.0 g/dL |
| Serum potassium | ≥3.5 mmol/L |

1. Subjects with a prior or concurrent malignancy whose natural history or treatment does not have the potential to interfere with the safety or efficacy assessment of the investigational regimen are eligible for this trial.
2. Subjects who are sexually active with a woman of childbearing potential must agree to use a condom with spermicidal foam/gel/film/cream/suppository and his partner must also be practicing a highly effective method of contraception (i.e., established use of oral, injected or implanted hormonal methods of contraception; placement of an intrauterine device or intrauterine system) during treatment and for 3-months following the last ADT treatment.
3. Ability to understand and the willingness to sign a written informed consent document.

## Exclusion Criteria

1. Subject has had prior or current PCa therapies, such as biologic, chemotherapy, hormone therapy, radiotherapy or surgery for PCa. Subjects may not have had undergone pelvic radiation, chemotherapy or immunotherapy treatment for a separate hematologic or visceral malignancy within 6-months of enrollment in the present study.
2. Subjects with locally advanced, nodal or metastatic prostate cancer.
3. Subjects who are unfit for pelvic mpMRI scanning (e.g., severe claustrophobia), permanent cardiac pacemaker, metallic implants that are likely to contribute to significant image artifacts, allergy or contraindication to gadolinium contrast agent.
4. History of allergy or intolerance to study drug components.
5. History of bilateral orchiectomy.
6. If the subject has an uncontrolled or major debilitating inter-current illness.
7. Subjects who are receiving any other investigational agents, or who have received other investigational agents in the past and who are no longer receiving these investigational agents may be eligible at the discretion of the principal investigator (PI).
8. Judgment by the treating investigator or PI that the subject is unsuitable to participate in the study and the subject is unlikely to comply with study procedures, restrictions, and requirements.

## Inclusion of Women and Minorities

Women will be excluded as the population under study is male. Minorities will be included.

# SCREENING & REGISTRATION PROCEDURES

## General Guidelines

Prior to registration and any study-specific evaluations being performed, all patients must have given written informed consent for the study and must have completed the pre-treatment evaluations. Patients must meet all of the eligibility requirements listed in [Section 5](#_PATIENT_SELECTION:_ELIGIBILITY). Eligible patients will be entered on study centrally by the University of Chicago study coordinator. All sites should contact the study coordinator at [PhaseIICRA@medicine.bsd.uchicago.edu](mailto:PhaseIICRA@medicine.bsd.uchicago.edu) to verify availability of a slot.

Following registration, patients must begin protocol treatment within 28 days. Issues that would cause treatment delays should be discussed with the Study Lead Principal Investigator. If a patient does not receive protocol therapy following registration, the patient’s registration on the study will be canceled. The study coordinator/CRA should be notified of cancellations as soon as possible.

## Registration Procedures

When a potential patient has been identified, notify the CRA via email at [PhaseIICRA@medicine.bsd.uchicago.edu](mailto:PhaseIICRA@medicine.bsd.uchicago.edu) to ensure a reservation on the study. Reservations for potential subjects will only be held for subjects who have signed consent for that particular study.

When registering a subject, the following must occur:

- - - Confirm that the institution has a current IRB approval letter for the correct version of protocol/consent and has an annual update on file, if appropriate.
    - Submit all required materials (Eligibility Checklist, Source documentation, and signed consent form) to confirm eligibility and required pre-study procedures to the CRA a minimum of 48 hours prior to the subject’s scheduled therapy start date.
    - Source documentation includes copies of all original documents that support each inclusion/exclusion criteria. The eligibility checklist does not serve as source documentation but rather as a checklist that original source documentation exists for each criterion.
    - Communicate with the CRA to ensure all necessary supporting source documents are received and the potential subject is eligible to start treatment on schedule. If there are questions about eligibility, the CRA will discuss it with the Study Lead PI. The Study Lead PI may clarify, but not overturn, eligibility criteria.
    - Affiliate sites must confirm registration of subjects by obtaining a subject study ID number from the CRA via phone, fax or email.
    - If a subject does not start on the scheduled day 1 treatment date, promptly inform the CRA as the delay in start may deem the subject ineligible and/or require further or repeat testing to ensure eligibility.
    - If randomization is involved, the date the patient is randomized will be considered the patient’s “On Study Date.” If randomization is not involved, the first time the patient receives treatment will be considered the patient’s “On Study Date.” The patient’s subject ID will be assigned and a confirmation of registration will be issued by the CRA on this date. Subjects that sign consent and do not go “On Study” will be recorded in the database with the date they signed consent and the reason for not going “OnStudy” (e.g., Ineligible, Screen Failure or Withdrawn Consent).

## Assignment of Screening and Subject Study Numbers

All consented subjects will be given a unique screening number that will be used to identify the subject for all procedures that occur prior to eligibility being confirmed. Each subject will be assigned only one screening number. Screening numbers must not be re-used for different subjects. The screening number will become the subject’s study number once they are confirmed to be eligible (i.e., registered for the clinical trial).

Any subject who is re-screened will be provided with a new screening number for each instance for which they are being screened. The screening number will be their study number as well once they are confirmed to be eligible and registered for treatment.

## Screen Failures & Re-Screening

Patients who are screen-failures may be re-screened at a later time to determine if they could meet eligibility criteria as long as they have not yet started treatment. The cost of re-screening tests will not be covered by the study. Any patient undergoing such re-screening must undergo informed consent and be provided with a new screening number for each instance for which they are being re-screened. Results from assessments performed during the prior screening period are acceptable in lieu of repeat screening tests if these prior tests are still within the protocol specified time frames. All screen-failures must be recorded in the study electronic data capture (EDC), REDCap.^27, 28^

## Subjects who do not undergo Focal Therapy or complete Focal Therapy

Any subject who has already received ADT and who for any reason is no longer a candidate for FT (e.g., if ablation of CSPCa cannot be done without damaging both neurovascular bundles, or patient withdraws consent for the procedure, or procedure is unfeasible for other reasons) the subject will be considered to be a “withdrawal” from treatment rather than a screen-failure. In the event FT is not performed or completed it will be up to the discretion of the treating investigator whether a subject should continue ADT (e.g., if the subject is receiving clinical benefit).

## Screening Prostate Cancer Localization by Multiparametric MRI

The mpMRI is a non-invasive radiologic investigation to identify prostate lesions amenable to FT. This pre-treatment imaging will already have been performed prior to being enrolled in the study. Upon screening evaluation of the subject’s mpMRI, no evidence of EPE and no evidence of seminal vesicle invasion is required for study enrollment. EPE will be evaluated using the 5-point Likert scale:

1 = EPE absent (normal tissue can be visualized between intact prostate capsule and tumor),

2 = EPE probably not present (tumor abuts prostate capsule),

3 = Equivocal for EPE (tumor abuts and causes irregularity on prostate capsule),

4 = EPE probably present (tumor bulges, deforms, and obscures the prostate capsule),

5 = EPE absolutely present (gross and measurable tumor is identified).

Accordingly, any scale of 1, 2, or 3 lesion will be negative for EPE, while any scale 4 or 5 lesion will be positive for EPE.

## Screening Prostate Cancer Diagnosis by Systematic and MRI Targeted Prostate Biopsy

The initial biopsy used to diagnose the subject’s PCa will already have been performed before the screening visit. Both the transperineal and transrectal approaches for MRI-TB are allowed for the present study. Of note, for the purposes of the present study, the subject’s MRI-TB is required to have a minimum 12-core systematic (random) prostate biopsy in addition to the targeted biopsy (2 or more cores per target lesion) of the suspicious lesion(s) seen on prostate mpMRI. Three-dimensional data on the location and specific grade for each lesion will be available and necessary to facilitate FT planning.

Patients will be considered candidates for FT if, following their MRI-TB, they have CSPCa (GG 2 or higher) corresponding to one or more mpMRI visible lesion(s) (PI-RADS ≥3) and ablative treatment would result in complete destruction of CSPCa while avoiding damage to the urethral sphincter and sparing at least one neurovascular bundle (left or right). Examples of potential FT templates to be used are described in [section 8.5.2](#_Focal_Therapy_Procedure).

The treatment or ablation zone will cover the area of the gland in which the CSPCa lesion(s) have been identified by the subject's MRI-TB. The lesion(s) may be derived from targeted cores, systemic cores, or both. Ablation will proceed as follows:

1. A maximum of 60-75% of the prostate may be ablated in order to include all CSPCa. If the patient has more than one focal lesion on one side, all CSPCa lesions will be destroyed.
2. Treatment may reach the urethra and may cross the midline anteriorly or posteriorly.

At least one neurovascular bundle must be avoided. This is achieved by ensuring a minimum distance of ablation zone border to the contralateral neurovascular bundle of 5 mm. If ablation of CSPCa cannot be done without damaging both neurovascular bundles, the patient will not be eligible for FT.

## Standard of Care Imaging for Eligibility

A radiologic and independent review by the PI will be performed of the subject’s CTAP and/or BS imaging prior to enrollment. If the subject has not undergone one or both of the abovementioned imaging studies then one or both will be ordered as SOC evaluation for unfavorable intermediate-risk and high-risk PCa. Any timeframe for these scans is acceptable so long as they are prior to enrollment.

## Timeframes for Screening Procedures

Results of a test performed prior to the subject signing consent as part of routine clinical management are acceptable in lieu of a screening test if performed within any protocol specified time frames. Screening procedures are to be completed **within 28-days prior** to the first dose of ADT except for the following:

- CTAP/ BS and/or PSMA PET scan must be obtained within 6-months prior to enrollment.
- The MRI-TB used for initial diagnosis must not be obtained **greater than 1-year from the date of consent**.
- If the MRI-TB which initially diagnosed the subject’s PCa **was obtained greater than 3- months from the time of study consent** then a repeat PSA should be completed for screening to obtain a “baseline” PSA. This applies to all participants regardless of GG used for eligibility.
- All pre-treatment serum and urine studies must be obtained **within 15-days prior** to the first dose of ADT.
  - Pre-treatment serum studies include: a complete blood count (CBC), complete metabolic panel (CMP), and serum testosterone.

Subjects may be rescreened after initially failing to meet the inclusion/exclusion criteria as long as they have not yet started treatment with ADT; however, the cost of re-screening tests will not be covered by the study. Results from assessments performed during the initial screening period are acceptable in lieu of a repeat screening test if performed within the specified time frame and the inclusion/exclusion criteria is met.

# BIOMARKER, CORRELATIVE, AND SPECIAL STUDIES

## Biomarker Plan

**List of Biomarker Assays in Order of Priority**

| Priority | Biomarker Name | Biomarker Assay | Biomarker Type and Purpose | M/O | Timing | Specimen | Quantity Needed | Lab |
| --- | --- | --- | --- | --- | --- | --- | --- | --- |
| 1 | Banking for future study | Banking for future study | Banking for future study | O | At screening after eligibility, 6 wks after FT | whole blood in a purple-top EDTA tube | 3 ML | University of Chicago |
| 2 | Banking for future study | Banking for future study | Banking for future study | O | At FT,  12 month MRI-TB | 4 core biopsies | 4 core biopsies | University of Chicago |

**Specimen Collection Schedule**

| **Specimen Type** | **Baseline (After Eligibility confirmed but Pre-treatment)** | **FT** | **6 wks after FT** | **12 month MRI-TB** |
| --- | --- | --- | --- | --- |
| Single EDTA tube serum specimen | X |  | X |  |
| Core biopsies |  | X  Two 18-gauge biopsy cores will be collected from the target area(s) and one each from peritumoral and untreated prostate |  | X  Two 18-gauge biopsy cores from the ablated area(s) and one each from periablation zone and untreated prostate. |

## Correlative Studies

All samples will be labeled with a unique number. Patient identifiers will not be included on the samples and link between identifiers and unique number will be kept in secure database at each institution.

Serum and Biopsies banked for future research will be stored at the University of Chiago and will be anonymized. Any patient who withdraws may request destruction of remaining samples that have not yet been analyzed or anonymized; destruction will be performed per the standard operating procedures of the respective laboratories with which samples may be stored.

## Exploratory/Ancillary Correlative Studies

Serum and Biopsies will be banked for future research and will be stored at University of Chicago.

## Peripheral Blood or Tissue

Collection of Specimen(s):

- One 3mL EDTA tube at screening once eligibility is confirmed and at 6-months after FT

Handling of Specimens(s):

- Normal operating procedures; do not shake or freeze tubes; Label each tube as follows:
  - Clinical trial study number (GU2201)
  - Subject’s (ID) (site number, patient ID example: GU02-01)
  - Date the tube was drawn (example: 2/2/2018)
  - Time of blood draw (example: 15:00)
  - Study time-point (example: Day 1)

Site(s) Performing Correlative Study:

- University of Chicago for long term sample banking.

Shipping of Specimen(s):

- Please refer to relevant SOPs or guidance documents from UC for more details.
- Follow IATA shipping instructions and standards by properly labeling all shipping boxes to prevent delays.
- Attach provided FedEx Airbill to the shipping box.
- Ship per standard operating procedures; Notify [Lab TBD] ***the day of shipping the sample.***
- Samples to be shipped Monday-Thursday only via FedEx Priority Overnight.

Collection of Specimen(s):

- 4 core biopsies of prostate tissue at the time of focal therapy and at 6 month MRI-TB

Handling of Specimens(s):

- Normal operating procedures; Label each as follows:
  - Clinical trial study number using the UChicago
  - Protocol #
  - Subject’s (ID) (site number, patient ID number)
  - Date of collection (example: 2/2/2018)
  - Time of collection (example: 15:00)
  - Study time-point (example: FT)

Site(s) Performing Correlative Study:

- University of Chicago for long term sample banking.

Shipping of Specimen(s):

- Please refer to relevant SOPs or guidance documents from University of Chicago for more details.
- Follow IATA shipping instructions and standards by properly labeling all shipping boxes to prevent delays.
- Attach provided FedEx Airbill to the shipping box.
- Ship per standard operating procedures; Notify University of Chicago ***the day of shipping the sample.***
- Samples to be shipped Monday-Thursday only via FedEx Priority Overnight.

# TREATMENT PLAN

## Health-related Quality of Life Assessments

The following questionnaires will be obtained at the screening visit or if unable to be obtained at the screening visit, then at the initiation of ADT (see study calendar). These questionnaires are used to assess the subjects’ health-related quality of life (HRQoL):

1. **International Prostate Symptom Score (I-PSS)** the I-PSS provides a measure of urinary symptoms of benign prostatic hyperplasia. See Appendix C for more details on questionnaire scoring and instructions. Subjects will select the response for each question that most closely corresponds to their recent experiences.
2. **Sexual Health Inventory for Men (SHIM):** SHIM potency score assesses erectile function. See Appendix D for more details on questionnaire and scoring instructions. Subjects will select the response for each question that most closely corresponds to their recent experiences.
3. **Expanded Prostate cancer Index Composite Short Form (EPIC-26)**: a subject-validated PCa HRQoL instrument that measures a broad spectrum of urinary, bowel, sexual, and hormonal symptoms. See Appendix E for more details on questionnaire and scoring instructions. Subjects will select the response for each question that most closely corresponds to their recent experiences.

## Laboratory Testing

Each subject will have a “baseline” PSA value which must be the value obtained just prior to the MRI-TB which provided the initial histopathologic diagnosis. If the MRI-TB which initially diagnosed the subject’s PCa was obtained greater than 3-months from the time of study consent then a repeat PSA should be completed for screening to obtain a “baseline” PSA (unless one has been obtained for SOC at least 3-months after this initial biopsy in which case no repeat value is needed and this may be used for eligibility). This applies to all participants regardless of GG used for eligibility.

Baseline serum testosterone levels can be obtained at the time of the screening visit along with other screening labs if the patient does not have serum testosterone levels within 3-months of screening.

In addition to baseline, PSA and serum testosterone testing results will be collected for research purposes 8-weeks after initiation of neoadjuvant therapy and at 3-, 6-,9- and 12-months clinic visit following FT per SOC (see study calendar). The PSA values obtained at these timepoints are collected for SOC at normal clinical visits. After the 12-months study follow-up visit, patients will be followed-up on a schedule per SOC intervals.

If a subject shows an increase in PSA (>2 ng/mL higher than baseline PSA) at 8-weeks after initiation of neoadjuvant therapy, he will be considered to have biochemical progression and will be withdrawn from the study. He will be offered clinical care based on re-staging work-up per SOC.

## Pharmacological Treatments: Androgen Deprivation Therapy

Pharmacologic research treatment will be administered on an outpatient basis. No investigational or commercial agents or therapies other than those described below may be administered with the intent to treat the subject’s malignancy.

Subjects will initiate ADT via any generic luteinizing hormone-releasing hormone agonist such as leuprolide acetate 22.5 mg subcutaneous 3-month injection once at baseline for a total of 3-months of treatment. ADT will be dosed to complete 3-months of pharmacologic treatment per study protocol. Of note, each participant will complete at least 8-weeks of neoadjuvant treatment prior to FT.

## Multiparametric Magnetic Resonance Imaging 8-weeks Post-ADT Start & Identifying Focal Therapy Disease Targets

After ADT treatment have been initiated, it is anticipated that there will be a decrease in prostate volume due to this combination drug regimen. A change in prostate volume can affect the specific spatial location of a prostate lesion, and the lesion site may have reduced in volume or change morphologically in an unpredictable pattern (see Appendix B for imaging protocol). Ultimately, this study phenomenon can affect FT planning.

To identify if there have been any significant shifts in the size and or location of the previously identified prostate lesion(s) (as compared to the subject’s screening MRI-TB), due to the subject’s potential response to ADT. An additional mpMRI will be performed at 8-weeks after initiation of ADT but prior to FT. This 8-week mpMRI will be compared against the index mpMRI imaging used for eligibility into the study. This additional prostate mpMRI, similar to the mpMRI used for screening, is a radiologic non-invasive investigation.

This research specific 8-week (2-month) post-ADT mpMRI is necessary to ensure the treatment plan during FT is accurate, and also provides the best data to characterize the response to the proposed drug combination.

Pre-operative and all post-operative mpMRI imaging will be performed utilizing the SOC Prostate Imaging Reporting and Data System Version 2.1 (PI-RADS) (see Appendix B for details). Radiologists with expertise in pelvic mpMRI will identify the specified distribution of the lesion(s) and assign a PI-RADS score. Three-dimensional data on the location and specific PI-RADS score for each lesion will be marked by the radiologist and integrated into FT planning.

## Focal Therapy Procedure Combination with Androgen Deprivation therapy

Partial ablation of the prostate, although not considered to be standard among the intermediate- to risk PCa population, is a PCa management option for men interested in undergoing this treatment modality following the shared decision-making process with a urologist. Importantly, the description of the FT procedure that follows does not differ from partial ablation of the prostate provided for PCa patients as part of clinical care and is not considered in and of itself to be altered or changed in any way for research purposes.

Within 8- to 12-weeks of initiation of ADT, FT will be performed. The volume of tissue treated will be determined based on the discretion of the treating investigator, and as indicated according to the subject’s MRI-TB results at the time of his PCa diagnosis.

### Conduct of the Focal Therapy Procedure

FT will be performed by surgeon(s) with experience in this specific modality of PCa treatment at their respective institution, these persons(s) do not need to be members of the study team to perform this SOC procedure. FT is considered a clinical procedure, and thus the determination of and delivery of treatment to the ablation zone(s) is at the discretion of the treating investigator or operating surgeon. For the purposes of the present study, FT will be performed via cryoablation or HIFU ablation of the prostate. The choice of energy modality will be dependent on physician discretion, patient preferences and location of targeted treatment (posterior lesions for HIFU, cryoablation can be used in all locations).

### Treatment with Focal Therapy Procedure

Signed informed consent will be taken for FT. This will be an additional surgical consent. A phosphate enema will be administered on the night prior or morning of surgery to ensure an empty rectum. The type of anesthesia (regional or general) will be discussed with the patient and an anesthesiologist. The type of anesthesia chosen will aim to eliminate any patient movement during treatment to avoid any adverse complications. The patient will be placed in a dorsal lithotomy position. Appropriate weight-based heparin-based prophylaxis against any potential thrombo-embolic event will be used. Appropriate antibiotic prophylaxis will be given.

The FT procedure will be performed using the target(s) identified from the subject’s screening MRI-TB. For larger template ablation (i.e., hemiablation, hockey stick or anterior ablations; see diagrams below) or for cognitive targeting (operator ablates prostate tissue seen on live ultrasound that he/she recalls as the location of a lesion on the subject’s mpMRI should the operator choose to perform the procedure in this specific SOC fashion), the standard FT equipment without fusion of mpMRI and prostate ultrasound imaging will be used.

- Two 18-gauge biopsy cores will be collected from the target area(s) and one each from peri-tumoral and untreated prostate tissue for biobanking and exploratory outcomes on correlatives.

Cryoablation needles will be inserted to create an ice ball that covers both the mpMRI-identified prostate lesion and a 0.5-1 cm margin. One or more thermal sensing needles can be placed in following locations on surgeon’s discretion, anterior to the rectum and/or at the level of the external urethral sphincter to measure the temperature at respective sites. The thermal sensors anterior to rectum and at the level of urethral sphincter are placed to monitor temperature and avoid damage to these vital structures. Depending on lesion’s location relative to these structures the sensors may or may not be needed. After all of the needles have been placed, cystourethroscopy will be performed to rule out any needle placement through the urethra or bladder. If needles are found in such location(s), these needles will be taken out and repositioned followed by a repeat cystourethroscopy. Once it is ensured that there are no needles in urethra and bladder, the cystoscope will be withdrawn and a urethral warming catheter will be advanced into the bladder.

Cryoablation will then be initiated. The ice ball margin will be monitored using ultrasound imaging as it expands posteriorly. The thermal couplers will also be monitored to maintain adequate cryoablation temperature. Two freeze-thaw cycles will be performed. At the end of the procedure and once the cryoablation probes are removed perineal pressure will be held for approximately 5- to 10-minutes to achieve hemostasis. The urethral warming catheter will be left in for an additional 5-minutes and then exchanged for a Foley catheter. This Foley catheter will remain in place until the postoperative visit with in 1 week to have the catheter removed. The patient will be taken out of lithotomy position, extubated and returned to the post-anesthesia care unit.

To perform HIFU, the ablative ultrasound probe is first inserted into the patient’s rectum to visualize the prostate and attached to a stepper. A Foley catheter is then inserted under sterile conditions to allow for continuous bladder drainage and visualize the urethra. The prostate is then segmented and volume calculated. Foley catheter is then removed. Using patient’s MRI information, the treatment area will be planned to destroy the MRI visible area and surrounding 5-10 mm of normal tissue. Larger area of involvement will require template ablation such as hemiablation. The lesion and margin are then ablated with automated ultrasound energy. Care is taken to avoid injury to the most distal aspect of prostate apex, bladder neck, and the rectum. After completing the ablation the ablative ultrasound probe was taken out from the rectum. The patient is transported to the recovery room and discharged with an indwelling catheter. The catheter is then removed at an outpatient appointment in approximately 1-week.

**The following principles will dictate the ablation template**:

The treatment or ablation zone will cover the area of the gland in which the CSPCa lesion(s) (GG ≥2) have been identified by the subject's MRI-TB study as follows:

1. A maximum of 60-75% prostate ablation to include all CSPCa. If the patient has more than one focal lesion on one side, all CSPCa lesions will be destroyed.
2. Treatment may reach the urethra and may cross the midline anteriorly or posteriorly.
3. At least one neurovascular bundle must be avoided by ensuring a minimum distance of ablation zone border to the contralateral neurovascular bundle of 5 mm. If ablation of CSPCa cannot be done without damaging both neurovascular bundles, the patient will not be considered for FT.
4. Up to one redo FT treatment is permissible; if either the protocol’s 6-month MRI-TB result(s) of ablated/treated prostate tissue, or a ‘for-cause’ prostate biopsy (see [section 8.7](#_For_Cause_Tests)) are positive at any point in follow-up.

**The following diagrams illustrate some possible interventions using focal therapy: In each of these scenarios below contralateral nerves were spared.**


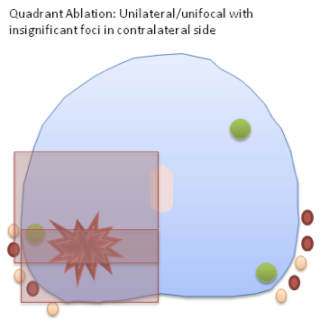

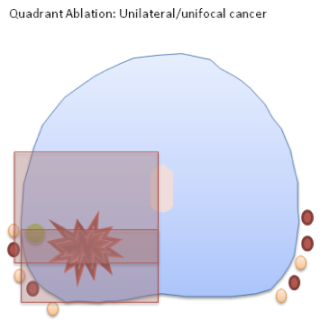


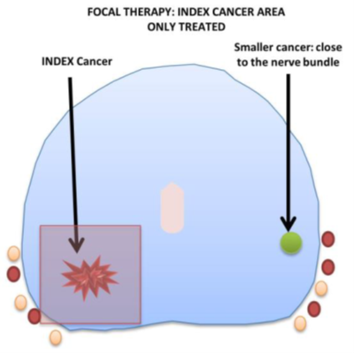


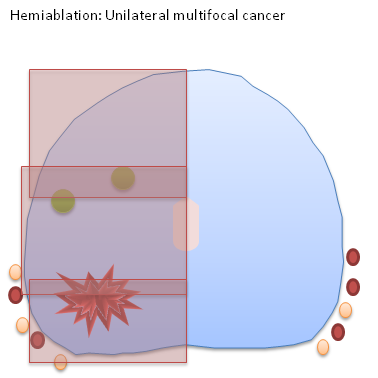


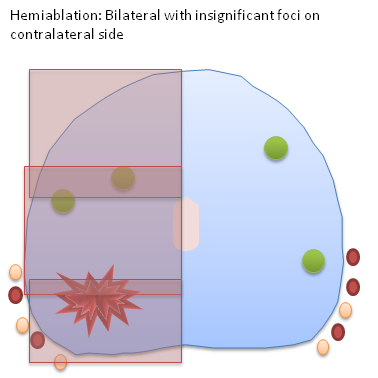


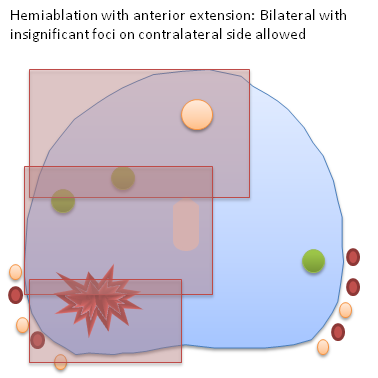


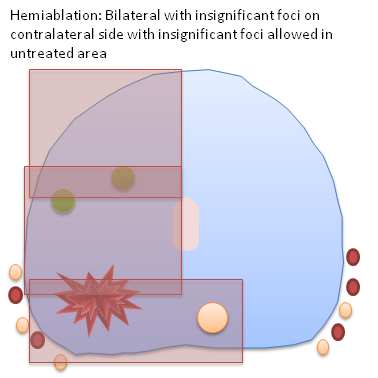


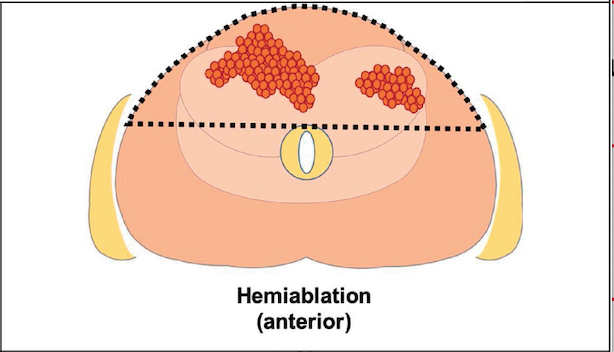


## Post-Focal Therapy Treatment Efficacy/Surveillance mpMRI and MRI-TB

At 12-months from the time of FT, a SOC mpMRI and MRI-TB will be performed to determine treatment response. Both the imaging study and prostate biopsy results will be used to measure efficacy of the proposed combination treatment. However, if a patient has any other post-treatment biopsy results these may also be used to assess for post-treatment efficacy even if not performed with an mpMRI guided biopsy.

Specifically, an MRI-TB will sample the ablated and unablated prostate tissue to determine if residual CSPCa (GG ≥2) remains in the treated and/or untreated prostate tissue. The mpMRI used for the MRI-TB will follow the same imaging protocol as the pre-treatment study and reported in the same manner. Documentation of the biopsy site(s) will be required. If a new suspicious radiographic lesion(s) is/are detected on the subject’s 12-month mpMRI which was/were not present in the pre-treatment [screening] mpMRI then the targeted biopsy cores of the suspicious area(s) will be obtained. Otherwise, if no suspicious lesions are noted in the 12-month mpMRI then random biopsies from the unablated prostate tissue will be obtained per SOC during the MRI-TB.

- Two 18-gauge biopsy cores will be collected from the ablated area(s) and one each from periablation zone and untreated prostate for biobanking and exploratory outcomes on correlatives.

If the 12-month MRI-TB identifies the presence of CSPCa in the ablated or unablated prostate tissue then the patient will be offered a second treatment with FT (if eligible for such treatments) and the follow-up visits for the re-treatment will be the same as the primary treatment. The patients will stay in the study and will continue to be followed up until 12-months from the first FT procedure. For patients with residual/recurrent cancer who decline or are ineligible for a second FT procedure, one of the whole gland treatments, either radical prostatectomy, whole gland ablation or radiation therapy, will be offered. These data will be recorded as part of report of secondary outcomes. Patients who choose to undergo whole gland treatment during the study follow-up will exit the study at this point. HRQOL surveys, PSA and testosterone levels will be obtained at the exit.

## For Cause Tests

Clinicians can biopsy the prostate following primary FT if there is a clinically significant rise in PSA (≥2 ng/mL higher from the subject’s post-FT PSA nadir, i.e., ‘for cause’ prostate biopsy) or clinical suspicion for progression of PCa. Nadir value will be defined as the lowest PSA value obtained after completion of combination treatment and recovery to non-castrate levels of testosterone (defined as testosterone levels >50 ng/mL). If there is a concern of prostate inflammation/infection due to a recent biopsy or urinary tract infection, patients will be treated with a course of antibiotics before repeating PSA. If the confirmatory PSA again demonstrates rise of 2 ng/mL above nadir, the patient will be evaluated further for local disease recurrence/progression. A mpMRI will be required prior to the biopsy unless the patient has developed a contraindication to biopsy.

If the untreated area is biopsied, the specimens will be individually identified according to location as determined by a standard protocol.

The specimen will be processed according to standard protocol and examined by a pathologist to note the following features for each core taken.

- Presence of adenocarcinoma
- Grade group
- Length of cancer and total core length

Additional Tests: ‘for cause’ additional tests such as ultrasound, CTAP, BS or PSMA/PET scan will be permissible as SOC practice.

# TOXICITY MANAGEMENT GUIDELINES/SAFETY MONITORING

## Falls and fractures

Monitor and manage patients at risk for fractures according to established treatment guidelines and consider use of bone-targeted agents.

## Ischemic heart disease and ischemic cerebrovascular disorders

Ischemic heart disease and ischemic cerebrovascular disorders, including events leading to death, occurred in patients treated with ADT. Monitor for signs and symptoms of ischemic heart disease and ischemic cerebrovascular disorders. Optimize management of risk factors, such as

hypertension, diabetes, or dyslipidemia. Of note, see screening exclusion criteria regarding patients with pre-existing heart disease for further clarification.

## General Concomitant Medication and Supportive Care Guidelines

The following concomitant medications and therapies are prohibited during the study. The Sponsor-Investigator must be notified in advance, or as soon as possible thereafter, of any instances in which prohibited therapies were administered. These must be reviewed for at study entry during screening.

- Any chemotherapy, anti-cancer therapy (other than study treatment[s]), or experimental therapy
- Radiotherapy to tumor lesions being assessed for tumor response prior to radiographic progression.

Subjects, who, in the assessment by the treating investigator, require the use of any of the aforementioned treatments for clinical management other than specified as allowed, should be removed from the trial. Subjects may receive other medications that the treating investigator deems to be medically necessary.

# DURATION OF TREATMENT, FOLLOW-UP & WITHDRAWALS

## Duration of Therapy

In the absence of treatment delays due to adverse event(s) (AE), treatment may continue per protocol until one of the following criteria applies:

- Disease progression
- Intercurrent illness that prevents further administration of treatment
- Unacceptable AE(s)
- Patient decides to withdraw from the study treatment
- General or specific changes in the patient's condition render the patient unacceptable for further treatment in the judgment of the treating investigator
- If a subject shows an increase in PSA (>2 ng/mL higher than baseline PSA) at 8-weeks after initiation of neoadjuvant therapy, he will be considered to have biochemical progression and will be withdrawn from the study.
- Patient non-compliance
- Termination of the study by University of Chicago PI

The reason(s) for protocol therapy discontinuation, the reason(s) for study removal, and the corresponding dates must be documented in the study EDC (REDCAP) and within study source documentation.

## Duration of Follow-Up

Subjects will be followed for survival and recurrence for up to 1-year from the date of their FT procedure. Subjects removed from study for unacceptable AE(s) will be followed until resolution or stabilization of the AE.

Refer to the study calendar (section 15) for the timing and frequency of the following activities in follow-up. Subjects in the post-op follow-up phase should be seen in clinic to collect PSA levels (per clinical SOC) and assess for survival and recurrence until death, withdrawal of consent, becoming lost to follow-up or at the end of the study (up to 1-year after FT procedure), whichever occurs first. HrQOLs (I-PSS, SHIM and the EPIC-26) will also be completed at designated follow-up visits. Subjects may also have a focused abdominal and pelvic genitourinary examination in follow-up if clinically indicated.

## Lost to follow-up

Patients who have begun but not completed active treatment with ADT who refuse to continue participation in the study, including any telephone contact, should be documented as “withdrawal of consent” rather than “lost to follow-up.”

In the absence of a clear withdrawal of consent, study teams should make every attempt to contact subjects during the post-FT follow-up phase to determine the patient’s survival or recurrence status or to collect other study data as needed following the University of Chicago Medicine Comprehensive Cancer Center (UCCCC) workflows or institution-specific requirements. A patient will be considered to be “lost to follow-up” per UCCCC workflows and policies or institution-specific requirements.

In the event that a subject has withdrawn consent to the collection of any follow-up data, the survival status of the patient can be obtained instead from publicly available death registries or other publicly available (e.g., obituaries) where it is possible to do so under applicable local laws to obtain a current survival status.

## Withdrawal of Consent

Subjects are free to withdraw from the study at any time. A subject who withdraws consent will always be asked about the reason(s) for withdrawal and the presence of any AEs. The PI and/or treating physician will continue to follow up AEs outside of the clinical study.

If a subject withdraws consent, they will be specifically asked if they are withdrawing consent to:

- All further participation in the study including any further follow up (e.g., contact for survival status, and HrQOL data collection in follow-up) and/or
- Only active treatment with ADT or the FT procedure.

The withdrawal of consent must be documented in REDCap and in source documentation.

When a subject is discontinued/withdraws to active treatment with ADT prior to completion per protocol, all applicable activities scheduled for the safety visit should be performed at the time of discontinuation. Any AEs, which are present at the time of discontinuation or withdrawal, should be followed in accordance with the safety requirements outlined in [Section 14](#_SAFETY_EVENT_AND).

# OTHER RESEARCH ACTIVITY SPECIFICATIONS

The Study Calendar summarizes the trial procedures to be performed at each visit.

## Medical History

A medical history for each subject will be obtained by the treating investigator or qualified designee. Medical history will include all active conditions, and any condition diagnosed within the prior 10-years that are considered to be clinically significant by the treating investigator. Details regarding the disease for which the subject has enrolled in this study will be recorded separately as part of a subject’s demographics and not listed as medical history.

Medical history must be graded per the National Cancer Institute’s Common Terminology Criteria for Adverse Events (CTCAE) v.5 to facilitate the identification of grade changes from baseline. Subjects will be asked about their medical conditions at each study visit (e.g., any new admissions or changes in existing conditions) and new medical history, if any, will be recorded throughout the study.

## Prior and Concomitant Medications

Prior Medications

The treating investigator or qualified designee will review prior medication use, including any protocol-specified washout requirements, and record prior medication taken by the subject within 28-days before starting the trial (time of consent). Treatment for the disease for which the subject has enrolled in this study will be recorded separately and not listed as a prior medication.

Concomitant Medications

The treating investigator or qualified designee will record all medications (including any supplements), if any, taken by the subject during the trial as specified in the Study Calendar (section 15) including those taken for any AEs.

## Adverse Events

The treating investigator or qualified designee will assess each subject to evaluate for potential new or worsening AEs as specified in the Study Calendar (section 15) or more frequently if clinically indicated. Please refer to Section 14 for detailed information regarding the assessment and recording and reporting of AEs.

## Full Physical Exam

The treating investigator or qualified designee will perform a full physical exam during the screening period. Clinically significant abnormal findings should be recorded as medical history. Full physical exam requires assessment of major organ sites (Constitutional, Head and Neck, Cardiovascular, Pulmonary, Abdominal, Musculoskeletal, Lymph, Neurological, and Skin).

## Focused Physical Exam

Except for at screening, the treating investigator or qualified designee will perform a focused abdominal and pelvic genitourinary physical exam during the baseline visit and at each follow-up visit post-operatively if clinically indicated.

## Vital Signs

The treating investigator or qualified designee will take vital signs at screening. Vital signs should include: temperature, pulse, respiratory rate, height, weight and blood pressure.

## Eastern Cooperative Oncology Group (ECOG) Performance Scale

The treating investigator or qualified designee will assess ECOG status **(see Appendix A)** at screening.

## Assessment of Disease

Only the treating investigator or qualified designee (MD only) may determine the assessment of disease recurrence or response. Response evaluation will be based on the I-PSS, the SHIM and the EPIC-26, and based upon the subject’s post-FT 6-month mpMRI & MRI-TB.

## Laboratory Safety Evaluations (Hematology, Chemistry and Urinalysis)

Laboratory tests for hematology, chemistry, and others are specified in Table 5 and should be performed at the timepoints specified in the study calendar.

**Table 5. Laboratory Tests**

| **Hematology** | **Chemistry** | **Other** |
| --- | --- | --- |
| Hematocrit | Albumin | PSA |
| Hemoglobin | Alkaline phosphatase | Testosterone |
| Platelet count | Alanine aminotransferase (ALT) |  |
| WBC (total and differential) | Aspartate aminotransferase (AST) |  |
| Red Blood Cell Count | CO_2_ or bicarbonate |  |
| Absolute Neutrophil Count | Calcium |  |
| Absolute Lymphocyte Count | Chloride |  |
| Absolute Eosinophil Count | Glucose |  |
|  | Blood Urea Nitrogen |  |
|  | Potassium |  |
|  | Sodium |  |
|  | Creatinine |  |
|  | Total protein |  |
|  | Total Bilirubin |  |
|  | Direct Bilirubin (If total bilirubin is elevated above the upper limit of normal) |  |

# STATISTICAL CONSIDERATIONS

## Study Design/Endpoints

This study will be a multi-institutional single arm phase I/II trial with a recruitment goal of 57 patients with treatment naïve non-metastatic intermediate-risk PCa.

Primary endpoints

1. To determine the proportion of men with CSPCa (GG ≥2) in the ablated prostate tissue by performing a surveillance mpMRI and MRI-TB at 12-months following FT.
2. To determine the proportion of men with CSPCa (GG ≥2) in the unablated prostate tissue by performing a surveillance mpMRI and MRI-TB at 12-months following FT.
3. To demonstrate the safety of combining ADT, and FT for the treatment of men with histopathologic diagnosis of GG 2 & 3 PCa, with PSA level < 20 ng/mL utilizing the NCI’s CTCAE v.5 classification to quantify and characterize the incidence of AEs.

Secondary endpoints

1. To define change in genitourinary and sexual function from baseline following ADT, and FT by measuring the subject’s HRQoL (I-PSS, SHIM, EPIC-26) at 6-and 12-months after FT.
2. To determine the PSA response to the combination treatment by measuring the subject’s PSA at “baseline” (PSA at time of initial diagnosis) and then at 3-months, 6-months, and 1-year from FT.
3. To determine the proportion of men converting to whole gland therapy (radical prostatectomy or radiation therapy) and/or requiring systemic therapy and/or developing metastases and/or dying of PCa during the course of study.
4. To determine the proportion of men without any PCa on any post treatment prostate biopsy.
5. To determine the proportion of men with normal baseline serum testosterone who had testosterone recovery (defined as testosterone levels >300 ng/dL) at 6-, 9- and 12-months after FT.

## Sample Size/Accrual Rate

It is estimated that the 30% of men who undergo FT for intermediate-risk PCa will have CSPCa (GG ≥2) on repeat biopsy after FT. We hypothesize the combination of ADT, and FT will halve the proportion of men with residual or recurrent CSPCa to 15%. Using sample size estimates for a single proportion, comparing a null proportion of 0.30 to an alternative proportion of 0.15, with an alpha = 0.05 and power at least 80%, the total number of patients required will be 51 men. We estimate 10% attrition for a total sample size of 57 men. We are expecting 18-21 months to enroll all the patients and 36-months to complete the study.

## Stratification Factors

None

## Analysis of Primary and Secondary Endpoints

Analysis Populations

Intent-to-Treat (ITT) Population

The ITT population includes all subjects who were enrolled and treated with the combination treatment (ADT (any duration) + FT). Note: Subjects who are enrolled but not treated will be documented as screen failures. The ITT population will serve as the primary analysis set for all safety and efficacy endpoints.

Per Protocol (PP) Population

The PP population includes all subjects who were enrolled and treated with the combination treatment (ADT (any duration) + FT), and who had no major protocol deviations. The PP population will serve as a supportive analysis set for all efficacy endpoints.

Missing Data

The following imputation methods will be used for the primary analysis of all endpoints:

• When calculating the proportion of subjects with a negative biopsy for CSPCa, subjects with missing biopsy information post-ablation will be imputed as “positive”

• Missing PSA data will be imputed using last observation carried forward (LOCF) (or previous median/mean or regression-based methods if proper).

• Missing data for IIEF-5, IPSS and EPIC questionnaires will be imputed using a multiple imputation approach at the item/question level.

All other endpoints/data (e.g., safety events) will be analyzed based on available (non-imputed) data, unless specified otherwise.

Baseline demographic and clinical information will be summarized using standard summary statistics, graphs and charts (see below).

**Descriptive tables as shown below will be populated:**

| **Baseline characteristics** |
| --- |
| Age (years), mean (SD, range) |
| Serum PSA (ng/ml), mean (SD, range) |
| Race |
| Reason for PSA test and Biopsy, % (n)  PSA screening (patient request)  Lower urinary tract symptoms |
| Prostate Volume (mL), mean (SD, range) |
| PSA Density (ng/mL^2^), mean (SD, range) |
| Grade group score on MRI-TB, % (N)  3+4  4+3 |
| MRI-TB, mean (SD, range)  Total Cores  Total Positive Cores  % Positive Cores |
| Risk category (%, n)  Unfavorable intermediate-risk |

| **Peri-operative characteristics** |
| --- |
| Procedure time (minutes), mean (SD, range) |
| Procedure type: HIFU or Cryoablation |
| Discharge time from procedure end (hours), mean (SD, range) |
| Length of catheterization (days), mean (SD, range) # |
| Dysuria (negative urine culture), % (n)  Duration (days), mean (SD, range) |
| Intermittent hematuria (start of stream only), % (n)  Duration (days), mean (SD, range) |
| Urinary debris, % (n)  Duration (days), mean (SD, range) |
| Urinary tract infection (positive urine culture) (%, n) |
| Stricture (%, n) |
| Recto-urethral fistula (%, n) |

| **Histological characteristics at 12-months MRI-TB** |
| --- |
| Number of cores taken, mean (SD, range) |
| PSA density (ng/mL^2^), mean (SD, range) |
| Absence of any cancer (%, n) |
| Absence of Gleason pattern 4 (%, n) |

The primary objective will be expressed as proportions of total number of patients and the 95% confidence interval of the proportion will be presented. Number and grade of complications will be reported.

HrQOL measures collected at 6- and 12-months will be compared to baseline measures using paired tests. In addition, the 6-month and 12-month change from baseline will also be tabulated

PSA: The mean PSA levels at each visit, the overall percent reduction in PSA levels from baseline to each visit, and the proportion of subjects with a PSA reduction compared to baseline at each visit will be presented. Mean PSA nadir and post-nadir PSA values through 12-months post-treatment will be summarized.

Testosterone recovery: Number of men who have recovered normal serum testosterone levels will be expressed as proportions of total number of eugonadal patients and the 95% confidence interval of the proportion will be presented.

Secondary or Adjuvant Treatment: The number and proportion of subjects undergoing secondary treatment during the study will be presented.

# SAFETY EVENT AND PRODUCT QUALITY COMPLAINTS REPORTING – PRINCIPAL INVESTIGATOR RESPONSIBILITIES

## Health Authority

As the Principal Investigator of the study and to the extent required by any applicable laws or regulations in each country/territory, the Institution/Investigator (University of Chicago/PI) shall be responsible for complying, within the required timelines, with any safety reporting obligation towards the competent health authorities, the Ethics Committees (EC) or Independent Review Board (IRB) and the participating (co- or sub-) investigators.

## General

Training: Institution/Sponsor of the Study shall be responsible for training the Study personnel (including the Investigator) on managing safety information arising from the Study according to agreed procedures and the requirements of this Agreement.

Maintenance of Safety Information: All safety data arising from the Study shall be maintained in a clinical database in a retrievable format. The Institution and Investigator shall provide a summary of all non-serious adverse events (NSAES) annually, and both serious and non-serious adverse events will be summarized in the final Study report.

## Adverse Event (AE) Definitions

- - 1. Adverse Events

An adverse event is any untoward medical occurrence in a patient or clinical investigation subject administered a pharmaceutical product that does not necessarily have a causal relationship with the treatment. An adverse event can be any unfavorable and unintended sign (including a laboratory finding), symptom or disease temporally associated with the use of a medicinal (investigational) product, whether or not related to the medicinal (investigational) product.

At each evaluation patients should be interviewed in a non-directed manner to elicit potential adverse reactions from the patient. The occurrence of an adverse event will be based on changes in the patient’s physical examination, laboratory results, and/or signs and symptoms, and review of the patient’s own record of adverse events.

Adverse events will be followed until resolution while the patient remains on-study. Once the patient is removed from the study, events thought to be related to the study medication will be followed until resolution or stabilization of the adverse event, or until the patient starts a new treatment regimen, or death, whichever comes first. Subjects will be followed for AEs/SAEs for 30 days after their last dose of study drugs.

- - 1. Unexpected Events

Unexpected events are those not listed at the observed specificity or severity in the protocol, informed consent, investigator brochure, or FDA-approved package insert. An event is considered unexpected if it is listed as occurring within the class of drugs or otherwise expected from the drug’s pharmacological properties but has not been previously observed with this specific investigational agent.

- - 1. Adverse Reactions

An adverse event is considered to be an adverse reaction if there is evidence to suggest a causal relationship to the study agent. This may include a single occurrence of an event strongly associated with drug exposure (e.g. Stevens-Johnson Syndrome), one or more occurrence of an event otherwise uncommon in the study population, or an aggregate analysis of specific events occurring at greater than expected frequency.

## Serious Adverse Event Definitions

### Serious Adverse Events

## Any adverse event occurring that:

- - Results in death
  - Is life-threatening
  - Requires in-patient hospitalization or prolongation of existing hospitalization
  - Results in persistent or significant disability/incapacity
  - Is a congenital anomaly/birth defect
  - Any suspected transmission of any infectious agent via administration of a medicinal product
  - Is considered medically significant*

*Any untoward medical occurrence that is considered medically significant. Medical and scientific judgment should be exercised in deciding whether expedited reporting is appropriate in other situations, such as important medical events that may not result in death, be life-threatening or require hospitalization but may be considered a serious adverse drug experience when, based on appropriate medical judgement, that may jeopardize the patient or subject and may require medical or surgical intervention to prevent one of the other outcomes listed in the bulleted list above. Examples of such medical events include allergic bronchospasm requiring intensive treatment in an emergency room or at home, blood dyscrasias or convulsions that do not result in hospitalization, or development of drug dependency or drug abuse or malignancy.

### Hospitalization

For reports of hospitalization, it is the sign, symptom or diagnosis which led to hospitalization that is the serious event for which details must be provided. Any event requiring hospitalization or prolongation of hospitalization that occurs during a study must be reported as a serious adverse event, except hospitalizations for the following:

- Hospitalizations not intended to treat an acute illness or adverse event (e.g., social reasons such as pending placement in long-term care facility)
- Surgery or procedure planned before entry into a study. [Note: Hospitalizations that were planned before the signing of ICF and where the underlying condition for which the hospitalization was planned has not worsened will not be considered serious adverse events. Any adverse event that results in a prolongation of the originally planned hospitalization is to be reported as a new serious adverse event.]

### Life-threatening Conditions

Disease progression should not be recorded as an adverse event or serious adverse event term; instead, signs and symptoms of clinical sequelae resulting from disease progression/lack of efficacy will be reported if they fulfil the serious adverse event definition.

## Adverse Event Reporting Requirements

- - 1. Routine Adverse Events Reporting

All Adverse Events must be reported in routine study data submissions. discussed in [Section 14.6.3](file:///C:\Users\samueltremblay\Desktop\FELLOW\Research\Dr.%20Sidana%20\Focal%20therapy%20-%20ADT%20protocol%20\Extraordinary#_) must also be reported in routine study data submissions.

- - 1. Serious Adverse Event Reporting to University of Chicago Medicine Comprehensive Cancer Center

Use the UCCCC protocol number and the protocol-specific patient ID assigned during trial registration on all reports.

All serious adverse events (as defined in [Section 14.4.1](#_Serious_Adverse_Event)) and all adverse events that have been specified to require expedited reporting occurring on this study require expedited reporting to the University of Chicago Comprehensive Cancer Center (UCCCC). The responsible Research Nurse or other designated individual at the treating site should report the SAE to the Study Lead Principal Investigator, the University of Chicago CRA and the Clinical Trial Support Office (CTSO) by the end of the business day when s/he becomes aware of the event. Events occurring after business hours should be reported to the CTSO by 12 p.m. (noon) the next business day. Reports should be made using the ‘Serious Event Report’ Form. Please scan and send via email (preferred) or fax to the following:

**University of Chicago Phase II CRA General:**

[PhaseIICRA@medicine.bsd.uchicago.edu](mailto:PhaseIICRA@medicine.bsd.uchicago.edu)

Phone:773-702-9885
Fax: 773-702-4889

**UCCCC Cancer Clinical Trials Office Quality Assurance:**

[qaccto@bsd.uchicago.edu](mailto:qaccto@bsd.uchicago.edu)

The completed form should be sent to the CTSO at [qaccto@bsd.uchicago.edu](mailto:qaccto@bsd.uchicago.edu) and to the Phase II CRA at [PhaseIICRA@medicine.bsd.uchicago.edu](mailto:PhaseIICRA@medicine.bsd.uchicago.edu) within the specified timelines above regardless of whether all information regarding the event is available. If applicable, a follow-up report should be provided to the regulatory team if additional information on the event becomes available.

All serious adverse events should also be reported to the local IRB of record according to their policies and procedures.

# STUDY CALENDAR

| **Visit number** | **1** | **2** | **3** | **4** | **5** | **6** | **7** | **8** | **9** |
| --- | --- | --- | --- | --- | --- | --- | --- | --- | --- |
| **Study milestones** | Screen | Drug Start | 8 wk mpMRI | FT | 30-Day EOT | 3 mo post-op | 6 mon  Post op | 9 mo  Post op | 1-year post-op  MRI-TB |
| **Time-point** | -28 days |  | 8 wks post V2 | 8-12 wks from V2 | 30-days post-FT | 3 mo post FT | 6mon-post-FT | 9 mo post FT | 1-year post-FT |
| **Windows** |  |  | +/- 7 days | +/-7 Days | +/- 7 Days | +/- 7 Days | +/- 2 Weeks | +/- 7 Days | +/- 4  Weeks |
| ADT^A^ |  | X |  |  |  |  |  |  |  |
| FT^B^ |  |  |  | X |  |  |  |  |  |
| Consent | X |  |  |  |  |  |  |  |  |
| Demographics | X |  |  |  |  |  |  |  |  |
| Med hx | X |  |  |  |  |  |  |  |  |
| Con meds^C^ | X | X------------------------------------X^C^ | | | |  |  |  |  |
| AEs^C^ | X | X-------------------------------------X^C^ | | | |  |  |  |  |
| Full PE | X |  |  |  |  |  |  |  |  |
| Focused PE^D^ |  |  |  |  | X | X | X | X | X |
| Vital signs^E^ | X |  |  |  |  |  |  |  |  |
| ECOG | X |  |  |  | X | X |  |  |  |
| I-PSS, SHIM^F^ | X |  |  |  | X | X | X | X | X |
| EPIC-26^F^ | X |  |  |  |  | X | X |  | X |
| PSA, Testosterone ^G^ | X^G^ |  | X |  |  | X | X | X | X |
| CBC/CMP/^H^ | X |  |  |  |  |  |  |  |  |
| mpMRI |  |  | X |  |  |  |  |  | X |
| MRI-TB |  |  |  |  |  |  |  |  | X |
| Correlative labs^I^ | X |  |  |  | X |  |  |  |  |
| Correlative bx^I^ |  |  |  | X |  |  | X |  | X |
| LTFU data^J^ |  |  |  |  | X | X | X |  | X |
| Remove FC^K^ |  |  |  | X^K^ |  |  |  |  |  |

**Calendar Footnotes**

1. ADT therapy will consist of any generic luteinizing hormone-releasing hormone agonist which will provide 3-months of treatment (e.g.,.
2. Focal therapy to be completed within 8- to 12-weeks of the initiation of ADT but after the completion of the 8-week mpMRI to allow for it to be used in FT planning.
3. Both adverse events (AEs) and serious AEs and any conmeds to be collected from the time of consent and continuously throughout the study until 30-days after the FT or as indicated in Section 14 of this protocol.
4. Focused abdominal and pelvic genitourinary exam. To be completed once a subject has entered into post-FT follow-up and only as clinically indicated.
5. Vital signs should include: temperature, pulse, respiratory rate, height, weight and blood pressure.
6. Health related quality of life measures including EPIC-26, the I-PSS and the SHIM must be collected at screening. If any of these are not done at screening then these may be completed at Visit 2.
7. PSA collected at “baseline” is defined within eligibility for this protocol. PSA and testosterone value is collected at 3-, 6-, 9- and 12-months post-FT.
8. Screening CBC & CMP, and testosterone must be collected and reviewed for safety within 15-days prior to the initial dose of ADT. Collection at other timepoints only if clinically indicated.
9. A single purple top EDTA tube of 3mL of whole blood to be collected after eligibility is confirmed for banking for future germline testing and at 6-weeks after FT. At the time of FT two 18-gauge biopsy cores will be collected from the target area(s) and one each from peritumoral and untreated prostate. At the time of 6-month MRI-TB two 18-gauge biopsy cores will be collected from the ablated area(s) and one each from periablation zone and untreated prostate.
10. Long term follow-up (LTFU) data collection to begin at 6-weeks after FT and includes collection of any recurrence, re-treatment, survival, and other relevant post-surgical data through 1-year post-FT.
11. FT foley catheter to be removed 1 weeks after FT +/- 3 days.

# MEASUREMENT OF EFFECT

For the purposes of this study, response evaluation will be based on the I-PSS, the SHIM and the EPIC-26.

# STUDY MANAGEMENT AND REGULATORY AFFAIRS

## Multicenter Guidelines

The specific responsibilities of the Study Lead Principal Investigator and the Coordinating Center are presented in Appendix B. Clinical studies coordinated by The University of Chicago must be conducted in accordance with the ethical principles that are consistent with Good Clinical Practices (GCP) and in compliance with other applicable regulatory requirements

The Study Lead PI/Coordinating Center is responsible for distributing all official protocols, amendments, and Safety Reports to all participating institutions for submission to their individual IRBs for action as required.

## Institutional Review Board (IRB) Approval and Consent

Unless otherwise specified, each participating institution must obtain its own IRB approval. It is expected that the IRB will have the proper representation and function in accordance with federally mandated regulations. The IRB should approve the consent form and protocol.

In obtaining and documenting informed consent, the treating investigator should comply with the applicable regulatory requirement(s), and should adhere to Good Clinical Practice (GCP) and to ethical principles that have their origin in the Declaration of Helsinki.

Before recruitment and enrollment onto this study, the patient will be given a full explanation of the study and will be given the opportunity to review the consent form. Each consent form must include all the relevant elements currently required by the FDA Regulations and local or state regulations. Once this essential information has been provided to the patient and the treating investigator is assured that the patient understands the implications of participating in the study, the patient will be asked to give consent to participate in the study by signing an IRB‑approved consent form.

Prior to a patient’s participation in the trial, the written informed consent form should be signed and personally dated by the patient and by the person who conducted the informed consent discussion.

## Required Documentation

Before the study can be initiated at any site, the following documentation must be provided to the Clinical Trials Support Office (CTSO) at the University of Chicago Comprehensive Cancer Center. All documents must be sent to CTSO prior to study activation.

- A copy of the official IRB approval letter for the protocol and informed consent
- Form FDA 1572 appropriately filled out and signed with appropriate documentation*)*
- Financial Disclosure Form for all investigators Data Safety Monitoring Committee

The UCCCC’s Independent Data and Safety Monitoring Committee (DSMC) will review and monitor study progress, toxicity, safety and other data from this study. Information that raises any questions about participant safety or protocol performance will be addressed by the Sponsor-Investigator, statistician and study team. Should any major concerns arise, the DSMC will offer recommendations regarding whether or not to suspend the study.

The DSMC will review the trial at least quarterly to review accrual, toxicity, response and reporting information. Information to be provided to the DSMC may include participant accrual; adverse events and serious adverse events; summary of any deaths on study; audit results; and a summary provided by the study team. Other information will be provided upon request.

## Trial Monitoring

This study will be remotely monitored by the University of Chicago CTSO Quality Unit Monitoring Staff in accordance with the standard operating procedure titled Monitoring of Multi-Institutional Investigator Initiated Clinical Trials.

Prior to subject recruitment, research staff will undergo a Site Initiation Teleconference to be conducted by the designated University of Chicago research team. The site’s Study Lead Principal Investigator and their study staff must attend the site initiation meeting.

Monitoring will be conducted to verify the following:

- - - - Adherence to the protocol
      - Completeness and accuracy of study data and samples collected
      - Compliance with regulations
      - Submission of required source documents

Research staff will also undergo a site close-out teleconference upon completion, termination or cancellation of a study to ensure fulfillment of study obligations during the conduct of the study, and to ensure that the Principal Investigator is aware of his/her ongoing responsibilities.

Protocol deviations are to be documented using the Protocol Deviation Form and sent via email to [PhaseIICRA@medicine.bsd.uchicago.edu](mailto:PhaseIICRA@medicine.bsd.uchicago.edu). Deviations that are considered major because they impact subject safety or alter the risk/benefit ratio, compromise the integrity of the study data, and/or affect subjects’ willingness to participate in the study must be reported within 7 days. Please contact the University of Chicago CRA ([PhaseIICRA@medicine.bsd.uchicago.edu](mailto:PhaseIICRA@medicine.bsd.uchicago.edu)) if you have questions about how to report deviations. All major protocol deviations should also be reported to the local IRB of record according to their policies and procedures.

## Auditing

In addition to the clinical monitoring procedures, the University of Chicago Comprehensive Cancer Center will perform routine Quality Assurance Audits of investigator-initiated clinical trials as described in the NCI-approved UCCCC DSM Plan. Audits provide assurance that trials are conducted and study data are collected, documented and reported in compliance with the protocol. Further, quality assurance audits ensure that study data are collected, documented and reported in compliance with Good Clinical Practices (GCP) Guidelines and regulatory requirements. The audit will review subjects enrolled at the University of Chicago in accordance with audit procedures specified in the UCCCC Data and Safety Monitoring plan. For institutions who are formal members of the CTSO Quality Unit, UCCCC will conduct on site quality assurance audits on average every two years during the enrollment and treatment phase of the study.

A regulatory authority (e.g. FDA) may also wish to conduct an inspection of the study, during its conduct or even after its completion. If an inspection has been requested by a regulatory authority, the site investigator must immediately inform the UCCCC Clinical Trials Support Office’s assigned study monitor that such a request has been made.

## Amendments to the Protocol

All modifications to the protocol, consent form, and/or questionnaires will be submitted to the University of Chicago IRB for review and approval. A list of the proposed modifications or amendments to the protocol and/or an explanation of the need of these modifications will be submitted, along with a revised protocol incorporating the modifications. **Only the Study Lead PI can authorize any modifications, amendments, or termination of the protocol.** Once a protocol amendment has been approved by the University of Chicago IRB, the UCCCC regulatory contact or their designee will send the amended protocol and consent form (if applicable) to the affiliate institutions electronically.

For external sites utilizing a local IRB:

- Upon receipt of the amendment documents the affiliate institution is expected to submit the amendment documents to their local IRB for approval as soon as possible.
- A copy of the IRB approval letter and approved consent document(s) should be sent to the UCCCC regulatory contract as soon as possible.
  - IRB approval should be obtained within 90 calendar days of the document distribution date. If approval cannot be obtained within this window, the reason for the delay should be provided to the designated UCCCC regulatory contact.

For external sites utilizing the BSD IRB as the IRB of record:

- Upon receipt of the amendment documents the affiliate institution is expected to implement the revised documents as soon as possible and no later than 30 calendar days from the document distribution date.
  - The date of local implementation should be documented in the local study records and provided upon request at time of monitoring and/or auditing.
- **No changes to the provided documents may be made at the local site without prior approval from the BSD IRB.**

## Annual IRB Renewals, Continuing Review and Final Reports

A continuing review of the protocol will be completed by the University of Chicago IRB and the participating institutions’ IRBs at least once a year for the duration of the study. The annual IRB renewal approvals for participating institutions should be forwarded promptly to the CTSO for filing. If the institution’s IRB requires a new version of the consent form with the annual renewal, the consent form should be included with the renewal letter.

## Record Retention

Study documentation includes all CRFs, data correction forms or queries, source documents, Study Lead Principal Investigator/treating Investigator correspondence, monitoring logs/letters, and regulatory documents (e.g., protocol and amendments, IRB correspondence and approval, signed patient consent forms).

Source documents include all recordings of observations or notations of clinical activities and all reports and records necessary for the evaluation and reconstruction of the clinical research study.

Government agency regulations and directives require that all study documentation pertaining to the conduct of a clinical trial must be retained by the study investigator. In the case of a study with a drug seeking regulatory approval and marketing, these documents shall be retained for at least two years after the last approval of marketing application in an International Conference on Harmonization (ICH) region. In all other cases, study documents should be kept on file until three years after the completion and final study report of this investigational study.

## Obligations of Study Site Investigators

The Study Site Principal Investigator is responsible for the conduct of the clinical trial at the site in accordance with Title 21 of the Code of Federal Regulations and/or the Declaration of Helsinki. The Study Site Principal Investigator is responsible for personally overseeing the treatment of all study patients. He/she must assure that all study site personnel, including sub-investigators and other study staff members, adhere to the study protocol and all FDA/GCP/NCI regulations and guidelines regarding clinical trials both during and after study completion.

The Study Site Principal Investigator at each institution or site will be responsible for assuring that all the required data will be collected and entered into the CRFs. Periodically, monitoring visits or audits will be conducted and he/she must provide access to original records to permit verification of proper entry of data.

# REFERENCES

1. Siegel, R. L., Miller, K. D., Fuchs, H. E. et al.: Cancer Statistics, 2021. CA Cancer J Clin, **71:** 7, 2021

2. Leyh-Bannurah, S. R., Karakiewicz, P. I., Pompe, R. S. et al.: Inverse stage migration patterns in North American patients undergoing local prostate cancer treatment: a contemporary population-based update in light of the 2012 USPSTF recommendations. World J Urol, **37:** 469, 2019

3. Mohler, J. L., Antonarakis, E. S., Armstrong, A. J. et al.: Prostate Cancer, Version 2.2019, NCCN Clinical Practice Guidelines in Oncology. J Natl Compr Canc Netw, **17:** 479, 2019

4. Onik, G., Vaughan, D., Lotenfoe, R. et al.: "Male lumpectomy": focal therapy for prostate cancer using cryoablation. Urology, **70:** 16, 2007

5. Fraser, J., Gill, W.: Observations on ultra-frozen tissue. Br J Surg, **54:** 770, 1967

6. Whittaker, D. K.: Mechanisms of tissue destruction following cryosurgery. Ann R Coll Surg Engl, **66:** 313, 1984

7. Hoffmann, N. E., Bischof, J. C.: The cryobiology of cryosurgical injury. Urology, **60:** 40, 2002

8. Duijzentkunst, D. A., Peters, M., van der Voort van Zyp, J. R. et al.: Focal salvage therapy for local prostate cancer recurrences after primary radiotherapy: a comprehensive review. World J Urol, **34:** 1521, 2016

9. de Castro Abreu, A. L., Bahn, D., Leslie, S. et al.: Salvage focal and salvage total cryoablation for locally recurrent prostate cancer after primary radiation therapy. BJU Int, **112:** 298, 2013

10. Valerio, M., Ahmed, H. U., Emberton, M. et al.: The role of focal therapy in the management of localised prostate cancer: a systematic review. Eur Urol, **66:** 732, 2014

11. Oishi, M., Gill, I. S., Tafuri, A. et al.: Hemigland Cryoablation of Localized Low, Intermediate and High Risk Prostate Cancer: Oncologic and Functional Outcomes at 5 Years. J Urol, **202:** 1188, 2019

12. Shah, T. T., Peters, M., Eldred-Evans, D. et al.: Early-Medium-Term Outcomes of Primary Focal Cryotherapy to Treat Nonmetastatic Clinically Significant Prostate Cancer from a Prospective Multicentre Registry. Eur Urol, **76:** 98, 2019

13. Tay, K. J., Polascik, T. J., Elshafei, A. et al.: Propensity Score-Matched Comparison of Partial to Whole-Gland Cryotherapy for Intermediate-Risk Prostate Cancer: An Analysis of the Cryo On-Line Data Registry Data. J Endourol, **31:** 564, 2017

14. Lebastchi, A. H., George, A. K., Polascik, T. J. et al.: Standardized Nomenclature and Surveillance Methodologies After Focal Therapy and Partial Gland Ablation for Localized Prostate Cancer: An International Multidisciplinary Consensus. Eur Urol, **78:** 371, 2020

15. Ahdoot, M., Lebastchi, A. H., Turkbey, B. et al.: Contemporary treatments in prostate cancer focal therapy. Curr Opin Oncol, **31:** 200, 2019

16. Ehdaie, B., Tempany, C. M., Holland, F. et al.: MRI-guided focused ultrasound focal therapy for patients with intermediate-risk prostate cancer: a phase 2b, multicentre study. Lancet Oncol, **23:** 910, 2022

17. Lindner, U., Trachtenberg, J., Lawrentschuk, N.: Focal therapy in prostate cancer: modalities, findings and future considerations. Nat Rev Urol, **7:** 562, 2010

18. Ahmed, H. U., Hindley, R. G., Dickinson, L. et al.: Focal therapy for localised unifocal and multifocal prostate cancer: a prospective development study. Lancet Oncol, **13:** 622, 2012

19. Ghafoor, S., Becker, A. S., Stocker, D. et al.: Magnetic resonance imaging of the prostate after focal therapy with high-intensity focused ultrasound. Abdom Radiol (NY), **45:** 3882, 2020

20. He, Y., Tan, P., He, M. et al.: The primary treatment of prostate cancer with high-intensity focused ultrasound: A systematic review and meta-analysis. Medicine (Baltimore), **99:** e22610, 2020

21. Dason, S., Wong, N. C., Allard, C. B. et al.: High-intensity Focused Ultrasound (HIFU) as salvage therapy for radio-recurrent prostate cancer: predictors of disease response. Int Braz J Urol, **44:** 248, 2018

22. Cao, J. Z., Su, R., Pan, J. F. et al.: The Use of High-Intensity Focused Ultrasound (HIFU) Plus 150mg Bicalutamide as First Line Salvage Therapy for Local Recurrent Prostate Cancer. Front Oncol, **11:** 705025, 2021

23. Marra, G., Dell'oglio, P., Baghdadi, M. et al.: Multimodal treatment in focal therapy for localized prostate cancer using concomitant short-term androgen deprivation therapy: the ENHANCE prospective pilot study. Minerva Urol Nefrol, **71:** 544, 2019

24. Shah, T. T., Ahmed, H., Kanthabalan, A. et al.: Focal cryotherapy of localized prostate cancer: a systematic review of the literature. Expert Rev Anticancer Ther, **14:** 1337, 2014

25. Donnelly, B. J., Saliken, J. C., Brasher, P. M. et al.: A randomized trial of external beam radiotherapy versus cryoablation in patients with localized prostate cancer. Cancer, **116:** 323, 2010

26. Chin, J. L., Ng, C. K., Touma, N. J. et al.: Randomized trial comparing cryoablation and external beam radiotherapy for T2C-T3B prostate cancer. Prostate Cancer Prostatic Dis, **11:** 40, 2008

27. Harris, P. A., Taylor, R., Thielke, R. et al.: Research electronic data capture (REDCap)--a metadata-driven methodology and workflow process for providing translational research informatics support. J Biomed Inform, **42:** 377, 2009

28. Harris, P. A., Taylor, R., Minor, B. L. et al.: The REDCap consortium: Building an international community of software platform partners. J Biomed Inform, **95:** 103208, 2019

# APPENDIX A PERFORMANCE STATUS CRITERIA

| ECOG Performance Status Scale | | Karnofsky Performance Scale | |
| --- | --- | --- | --- |
| Grade | Descriptions | Percent | Description |
| 0 | Normal activity. Fully active, able to carry on all pre-disease performance without restriction. | 100 | Normal, no complaints, no evidence of disease. |
|  |  | 90 | Able to carry on normal activity; minor signs or symptoms of disease. |
| 1 | Symptoms, but ambulatory. Restricted in physically strenuous activity, but ambulatory and able to carry out work of a light or sedentary nature (*e.g.*, light housework, office work). | 80 | Normal activity with effort; some signs or symptoms of disease. |
|  |  | 70 | Cares for self, unable to carry on normal activity or to do active work. |
| 2 | In bed <50% of the time. Ambulatory and capable of all self-care, but unable to carry out any work activities. Up and about more than 50% of waking hours. | 60 | Requires occasional assistance, but is able to care for most of his/her needs. |
|  |  | 50 | Requires considerable assistance and frequent medical care. |
| 3 | In bed >50% of the time. Capable of only limited self-care, confined to bed or chair more than 50% of waking hours. | 40 | Disabled, requires special care and assistance. |
|  |  | 30 | Severely disabled, hospitalization indicated. Death not imminent. |
| 4 | 100% bedridden. Completely disabled. Cannot carry on any self-care. Totally confined to bed or chair. | 20 | Very sick, hospitalization indicated. Death not imminent. |
|  |  | 10 | Moribund, fatal processes progressing rapidly. |
| 5 | Dead. | 0 | Dead. |

# APPENDIX B Prostate Multiparametric Magnetic Resonance Imaging and Transperineal or Transrectal Magnetic Resonance Imaging Targeted Prostate Biopsy Protocols

The mpMRI protocol: see Prostate Imaging Reporting and Data system version 2.1 ([PI-RADS version 2.1](https://www.acr.org/-/media/ACR/Files/RADS/Pi-RADS/PIRADS-V2-1.pdf?la=en))

Pre-operative and all post-FT imaging will be performed using either a 1.5 or 3 Tesla scanner and a pelvic phased array receiver, with or without an endorectal coil. A full protocol of T1 and T2 weighted turbo-spin echo images and a dynamic post gadolinium volume acquisition will be used for both pre-operative diagnostic and planning scans and post-operative assessment of ablation. The protocol will be standardized as below:

- T1 weighted axial of pelvis: to detect lymphadenopathy and other pelvic
- pathologies
- T2 axial small field of view prostate: cancer detection and volume estimation
- T2 small field of view coronal: for cancer detection and to accurately determine the position of prostatic apex and external sphincter
- T1 axial small field of view: to detect hemorrhage
- Dynamic contrast-enhanced: for tumor enhancement.
- Post contrast small field of view fat saturated T1 axis: high-resolution images of enhancement
- Diffusion weighted axial scans of prostate: to detect restricted diffusion in tumor

**Transperineal or Transrectal Prostate Biopsy Protocols:**

The following will be used to report all transperineal template mapping biopsies:


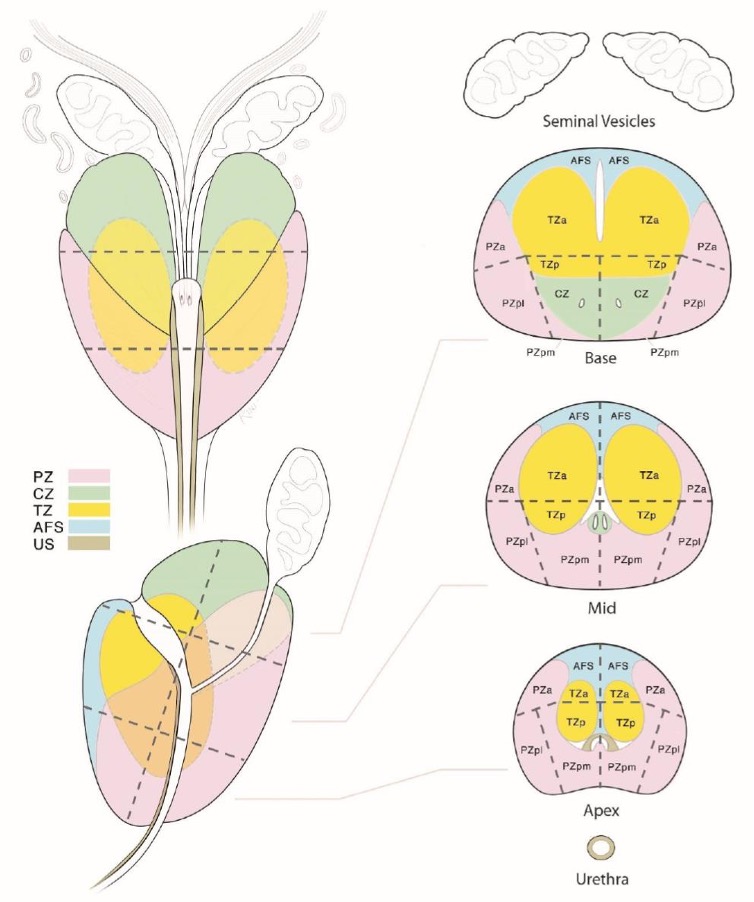


**Figure 1.1: PI-RADS v2.1 Sector Map**

Freehand or grid-assisted transperineal biopsy should include at least 2 targeted cores of any mpMRI visible lesion and systematic biopsy to include adequate sampling of the peripheral zone sectors (designated “PZpm, PZpl, PZa”) of PI-RADS v2.1 sector map. Systematic biopsies should include 10-14 cores with at least 2 cores from each peripheral zone sector with care taken to include both apical and basal sampling. In the event that the targeted lesion lies in one of the PI-RADS v2.1 peripheral zone sectors, systematic sampling from that sector may be omitted, but the systematic biopsy should include at least 2 cores from each peripheral zone sector not sampled with targeted biopsies.

The following format will be used to report transrectal template mapping biopsies:


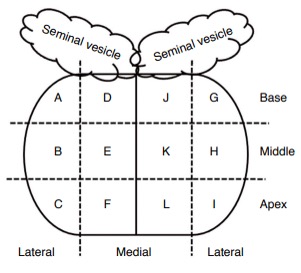


**Figure 1.2: Transrectal 12-core biopsy schematic**

Schematic diagram of 12-core transrectal needle biopsy analysis. The prostate gland is divided into 12 compartments. A, right lateral base; B, right lateral middle; C, right lateral apex; D, right medial base; E, right medial middle; F, right medial apex; G, left lateral base; H, left lateral middle; I, left lateral apex; J, left medial base; K, left medial middle; L, left medial apex.

Transrectal biopsy should include adequate (leave number up to PI discretion) targeted cores of any mpMRI visible lesion and 12 core systematic biopsy to include all regions defined in transrectal 12 core biopsy schematic (Figure 2.2). In the event that the targeted lesion lies in one transrectal 12 core biopsy compartment, systematic sampling from that sector may be omitted.

**Optional Multiparametric Magnetic Resonance Imaging Transrectal Fusion Image Registration for the Focal Therapy Treatment Planning Protocol**

If a mpMRI/TRUS fusion device is being used (e.g. ARTEMIS™, Eigen Inc., Grass Valley, CA), manual contouring will be performed on the prostate capsule and the boundary of visible lesions (suspected to be cancer-bearing tissue) using special-purpose software. The lesion and prostate capsule will be delineated by the radiologist and/or a trained urologist.

Firstly, a standard set-up procedure will be performed with the cryoablation or HIFU systems and TRUS devices, prior to FT treatment. The area to be treated will then be planned by the operating surgeon i.e., according to pre-operative mpMRI, Biopsy findings and pre-operative discussion between the operating surgeon and the uro-radiologist, by stating the HIFU treatment zone(s) or the intended grid reference placement of the cryoablation probes within the 5 mm brachytherapy grid. These grid references will be recorded at this stage.

Secondly, a 3D ultrasound volume file will be acquired and used to register the mpMRI and ultrasound images. The accuracy of the image alignment will be visually inspected by the surgeon and information of the registered location of the tumor saved to a file. The information acquired will be used to visually assess the extent of the planned treatment through comparison of grid references between TRUS and an aligned grid on the registration software. At this stage, the surgeon may adapt the treatment plan based on the information from the registration software. This may include a decrease or increase in treatment volume, through movement of the cryoablation probes within the brachytherapy grid or adjustment of the HIFU treatment zone(s), with the constraint that the final image-registration-informed treatment volume may not exceed a predefined maximum volume of tissue (according to the ‘dose-escalation’ protocol). The operating surgeon will be free to reject the information provided by using the registration software if (s)he believes that this may compromise the subject treatment in any way. In this case, the surgeon will record the reasons, which will be stored with the other data collected as part of the study. The updated grid references or HIFU treatment zone(s) will be recorded at this stage, if a change has been made to the treatment plan.

# APPENDIX C: Quality of Life I-PSS

PDF versions of this assessment will be made available to the respective study teams and site for submission to their respective IRBs’ of record and use with study subjects.

# APPENDIX D: Quality of Life – SHIM

PDF versions of this assessment will be made **available** to the respective study teams and site for submission to their respective IRBs’ of record and use with study subjects.

# APPENDIX E: Quality of Life – EPIC-26

PDF versions of this assessment will be made **available** to the respective study teams and site for submission to their respective IRBs’ of record and use with study subjects.

# 24 APPENDIX F MULTICENTER GUIDELINES

Responsibility of the Study Lead PI

- The Study Lead PI will be the single liaison with regulatory and data management staff, outside sponsor/s, , and funding agencies. The Study Lead PI is responsible for the coordination, development, submission, and approval of the protocol as well as its subsequent amendments. **The protocol must not be rewritten or modified by anyone other than the Study Lead PI**. There will be only one version of the protocol, and each participating institution will use that document. The Study Lead PI is responsible for assuring that all participating institutions are using the correct version of the protocol.
- The Study Lead PI is responsible for the overall conduct of the study at all participating institutions and for monitoring its progress. All reporting requirements are the responsibility of the Study Lead PI.
- The Study Lead PI is responsible for the timely review of Adverse Events (AE) to assure safety of the patients.
- The Study Lead PI will be responsible for the review of and timely submission of data for study analysis.

Responsibilities of the Coordinating Center

- The Coordinating Center is responsible for maintaining copies of IRB approvals from each participating site.
- The Coordinating Center is responsible for central patient registration. The Coordinating Center is responsible for assuring that IRB approval has been obtained at each participating site prior to the first patient registration from that site.
- The Coordinating Center is responsible for the preparation of all submitted data for review by the Study Lead PI.
- The Coordinating Center will maintain documentation of AE reports. The Coordinating Center will submit AE reports to the Study Lead PI for timely review.
